# Modulation of lysine methylation in myocyte enhancer factor 2 during skeletal muscle cell differentiation

**DOI:** 10.1093/nar/gkt873

**Published:** 2013-09-27

**Authors:** Jinmi Choi, Hyonchol Jang, Hyunsoo Kim, Jong-Hyuk Lee, Seong-Tae Kim, Eun-Jung Cho, Hong-Duk Youn

**Affiliations:** ^1^Department of Biomedical Sciences and Biochemistry and Molecular Biology, National Creative Research Center for Epigenome Reprogramming Network, Ischemic/Hypoxic Disease Institute, Seoul National University College of Medicine, Seoul 110-799, ^2^Division of Cancer Biology, Research Institute, National Cancer Center, Goyang 410-769, ^3^Department of Molecular Cell Biology, Sungkyunkwan University School of Medicine, Suwon, ^4^National Research Laboratory for Chromatin Dynamics, College of Pharmacy, Sungkyunkwan University, Suwon 440-746 and ^5^WCU Department of Molecular Medicine & Biopharmaceutical Sciences, Graduate School of Convergence Science, Seoul National University, Seoul 110-799, Republic of Korea

## Abstract

Myocyte enhancer factor 2 (MEF2) is a family of transcription factors that regulates many processes, including muscle differentiation. Due to its many target genes, MEF2D requires tight regulation of transcription activity over time and by location. Epigenetic modifiers have been suggested to regulate MEF2-dependent transcription via modifications to histones and MEF2. However, the modulation of MEF2 activity by lysine methylation, an important posttranslational modification that alters the activities of transcription factors, has not been studied. We report the reversible lysine methylation of MEF2D by G9a and LSD1 as a regulatory mechanism of MEF2D activity and skeletal muscle differentiation. G9a methylates lysine-267 of MEF2D and represses its transcriptional activity, but LSD1 counteracts it. This residue is highly conserved between MEF2 members in mammals. During myogenic differentiation of C2C12 mouse skeletal muscle cells, the methylation of MEF2D by G9a decreased, on which MEF2D-dependent myogenic genes were upregulated. We have also identified lysine-267 as a methylation/demethylation site and demonstrate that the lysine methylation state of MEF2D regulates its transcriptional activity and skeletal muscle cell differentiation.

## INTRODUCTION

Chromatin-modifying enzymes regulate gene expression by modifying histones and interacting with master transcription factors (1). EHMT2/G9a is a histone methyltransferase that mediates mono- and dimethylation of histone H3K9 in euchromatic regions (2). G9a also targets many nonhistone proteins to control transcriptional activities during cell fate decisions and cellular responses to environmental stressors (2). For instance, G9a has been implicated in embryonic development, based on the embryonic lethality of G9a knockout mice (3). The regulation of G9a function affects the generation of induced pluripotent stem cells (iPSCs), and H3K9me2 is dynamically controlled during stem-cell differentiation (4,5).

The myocyte enhancer factor 2 (MEF2) family of transcription factors, which comprises four members (A–D), mediates several processes, including the differentiation, proliferation, survival and apoptosis of various cell types (6–9). Particularly during muscle differentiation, MEF2 targets downstream myogenic genes and is regulated over time and by location (8,10,11). Thus, to modulate MEF2 activity and effect its precise regulation of target genes, corepressors and coactivators are recruited to MEF2 target promoters. Calcineurin-binding protein-1 (Cabin1) recruits histone methyltransferases and deacetylases, such as Suv39h1 and HDACs, to repress MEF2 activity through chromatin remodeling (12–16).The histone demethylase LSD1 and acetyltransferase p300 activate MEF2 transcriptional activity by modifying the histones in MEF2 target promoters (17,18). Moreover, a histone chaperone, HIRA, in cooperation with Asf1, stimulates MEF2 transcriptional activity during muscle differentiation (19).

MEF2 activity is also regulated by posttranslational modifications, including sumoylation, phosphorylation and acetylation. Several kinases, including mitogen-activated protein kinase p38 and extracellular signal-regulated kinase 5 (ERK5), phosphorylate MEF2 to modulate its transcriptional activity (9,20,21). Moreover, acetylation at several sites in MEF by p300 and deacetylation by HDAC3 regulate such activity (22–24).

Although many regulatory mechanisms have been suggested to govern its function, how MEF2 regulates an extensive array of target genes during complex cellular processes remains unknown (25–27). Thus, we examined lysine methylation as a novel regulatory mechanism that enables MEF2 to orchestrate the expression profiles of target genes.

We report that MEF2D is methylated and demethylated by G9a and LSD1, respectively, which effects the dynamic regulation of MEF2D transcriptional activity and the expression of its target genes during skeletal muscle differentiation. During myogenic differentiation, MEF2D dissociates from G9a, and its methylation is reduced, upregulating myogenic genes that are targeted by MEF2D. Conversely, aberrant MEF2D methylation by overexpression or knockdown of G9a results in the dysregulation of muscle cell differentiation, implicating MEF2D as a master regulator in this process.

## MATERIALS AND METHODS

### Cell culture and transient expression

The C2C12 mouse myoblast cells and HEK 293 cells have been described (17). Polyethylenimine (PEI, Polysciences, Inc.) was used to transfect HEK293 cells. C2C12 cells were electroporated with the Neon Transfection System (Invitrogen) per the manufacturer’s instructions. Plat-E cells, E14 cells (28) and DO11.10 cells have been described (12).

### DNA constructs

Flag-MEF2D was generated by subcloning the HindIII-XhoI-digested PCR products from Myc-tagged MEF2D into pcDNA3.0/Flag (Invitrogen). HA-MEF2D, HA-MEF2D (1–130) and Myc-MEF2C have been described (17). pRSET(B)-MEF2D was generated by subcloning the XhoI-HindIII-cut PCR products from Myc-tagged MEF2D into pRSET(B) (Invitrogen). pCAG-MEF2D was generated by subcloning the XhoI-digested PCR product from HA-MEF2D into pCAG-IP or pMIG (Addgene) (28). Flag-G9a has been described (29). PCR products of truncated mutants of G9a were obtained from full-length G9a and inserted into pSG5-Flag. pMIG-G9a and pMSCV-G9a were generated by subcloning the EcoRI-digested PCR products from Flag-G9a into pMIG or pMSCV (Clonetech).

### Antibodies and reagents

BIX01294 was purchased from Santa Cruz and pargyline was purchased from Sigma. Anti-Flag (M2) and anti-G9a were purchased from Sigma; anti-Myc (9E10) and anti-HA (16B12) were obtained from Covance; anti-methyl lysine and anti-G9a were purchased from Abcam; anti-Ezh2 was obtained from Cell Signaling; anti-MEF2, anti-MHC and anti-myogenin were from Santa Cruz and anti-MEF2D was from BD Biosciences. ImmunoPure® Goat Anti-Mouse IgG, (H + L) and ImmunoPure® Peroxidase-Conjugated Goat Anti-Rabbit IgG, (H + L) were purchased from Pierce, and anti-mouse Alexa 488, anti-rabbit Alexa 568 (Molecular Probes) and DAPI were obtained from Calbiochem. Anti-K267me was generated from Abmart by immunization with 263-APSR(meK)PDLR-271. Unmodified and mono-methyl-K267 MEF2D peptides were synthesized chemically (Abmart).

### Immunoprecipitation and reporter gene assay

Immunoprecipitation and reporter gene assays were performed as described (17). For the reporter gene assays using pOF-MEF2-luc, containing multimerized MEF2-binding sites and p*Myogenin*-luc, HEK293 cells were transfected with a *luciferase* reporter plasmid with HA-MEF2D and Flag-G9a. Cells were harvested 48 h after transfection, and luciferase activity was measured with an Infinite M200 (Tecan Group Ltd.).

### Retroviral infection

Empty or G9a-expressing viral vectors were transfected into the packaging cell line plat-E cells with Lipofectamine Plus (Invitrogen). Viral supernatants were harvested and used to infect C2C12 cells. Cells that were infected with pMSCV or pMSCV-G9a were selected with puromycin (4 μg/ml) for 2 days before use.

### Lentivirus production

To knock down G9a, lentiviral vectors that contained the mouse G9a-targeting sequences pLKO.1-sh-G9a #1 (TRCN0000054543) and #2 (TRCN0000054545) were purchased from Open Biosystems. pLKO.1 was used as a control. Lentivirus was produced per the manufacturer’s protocol using the BLOCK-iT Lentiviral RNAi Expression System (Invitrogen). Twenty-four hours after lentiviral infection, infected cells were selected with puromycin (4 μg/ml) for 2 days and used for experiments. pLKO.1-shG9a #2 was more effective and used all subsequent experiments. Knockdown of LSD1 has been described (17).

### Immunofluorescence

C2C12 cells were immunostained and observed as described (17).

### *In vitro* methylation and demethylation assay

Methylation assay was performed as described (30) using bacterially purified GST-G9a and His-MEF2D. Demethylation assay was performed as described (31) using bacterially purified GST-LSD1.

### ESI-LC-MS analysis

MEF2D was immunoprecipitated and separated on SDS–polyacrylamide gels. Gels were stained with Coomassie blue. Sliced gel pieces or MEF2D peptides were digested with trypsin or chymotrypsin and analyzed by ESI-LC-MS (Diatech Korea, Co. LTD).

### Quantitative real-time PCR and chromatin immunoprecipitation assay

ChIP and quantitative real-time polymerase chain reaction (qRT–PCR) were performed as described (17). Primers for RT–PCR and the position and sequence of the primers that were used to amplify ChIP-enriched DNA that spanned the MEF2-response elements have been described (17). Specific primers for qRT–PCR are listed in Supplementary Table S1. Primers for the mouse *Gapdh* promoter were 5′-GCACAGTCAAGGCCGAGAAT-3′ and 5′-GCCTTCTCCATGGTGGTGAA-3′.

### Statistical analysis

Data in the bar graphs were expressed as mean and standard deviation of three independent experiments. *P*-values were calculated using a student’s *t*-test calculator (http://www.physics.csbsju.edu/stats/t-test.html). *P* < 0.05 was considered statistically significant. All data are representative of at least three independent experiments.

## RESULTS

### MEF2D is methylated at K267

The regulation of MEF2 by various posttranslational modifications has been reported (8). However, lysine methylation of MEF2 and its effects on MEF2-dependent transcription during muscle differentiation have not been examined. Thus, we determine whether MEF2 is methylated at lysine residue(s) using MEF2D.

By immunoprecipitation of overexpressed MEF2D with anti-methyl lysine, MEF2D is methylated at a lysine residue (Supplementary Figure S1a). Methylation of overexpressed MEF2D in HEK293 cells was confirmed by ESI-LC-MS. A molecular shift (+28 Da) of the modified peptide (left upper panel) compared with the unmodified peptide (lower left panel) indicated dimethylation of lysine 267 (K267) of MEF2D (Supplementary Figure S1b). Furthermore, monomethylated MEF2D was detected by ESI-LC-MS (right panel) (Supplementary Figure S1b). Endogenous MEF2D from C2C12 mouse myoblast cells was also methylated at K267 by ESI-LC-MS (Supplementary Figure S1c).

**Figure 1.**
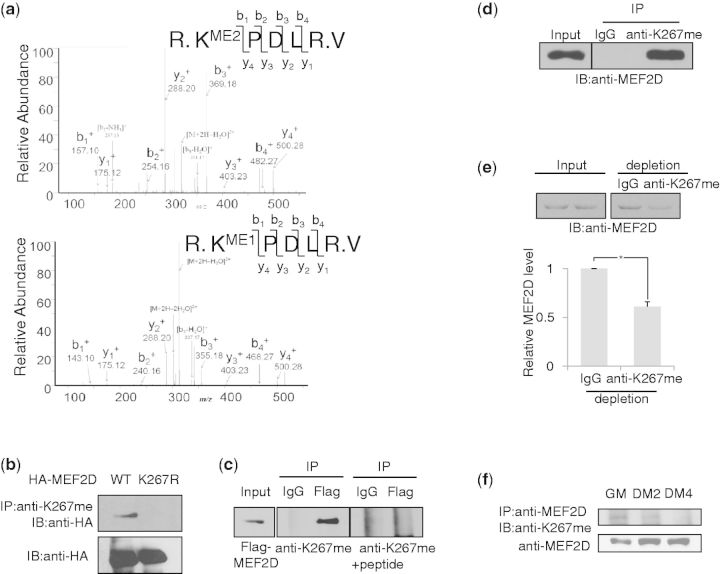
MEF2 methylation decreases during C2C12 cell differentiation. (**a**) HA-MEF2D, transiently expressed in HEK293 cells, was immunoprecipitated with anti-methylated K267 MEF2D (anti-K267me) and subjected to ESI-LC-MS analysis. Dimethylation (upper panel) and monomethylation (lower panel) of MEF2D were detected. (**b**) Transiently expressed HA-MEF2D wild-type (WT) or K267R mutant (K267R) was immunoprecipitated with anti-K267me, followed by immunoblotting with anti-HA. (**c**) Transiently expressed Flag-MEF2D (WT) was immunoprecipitated with anti-Flag, followed by immunoblotting with anti-K267me with or without chemically methylated K267 containing peptide blocking. (**d**) Endogenous MEF2D was immunoprecipitated from C2C12 cells with anti-K267me and immunoblotted with anti-MEF2D. (**e**) C2C12 whole-cell lysates were immunoprecipitated with anti-K267me or IgG. Supernatants of immunodepleted C2C12 cell lysates were analyzed by immunoblotting with anti-MEF2D (upper panel). Quantification of MEF2D after normalization to input (lower panel). (**f**) C2C12 cells, differentiated for up to 4 days, were immunoprecipitated with anti-MEF2D and western blotted with anti-K267me (GM, DM2, DM4; DM for 2 or 4 days).

To examine MEF2D methylation, a specific antibody for MEF2D with methylated K267 was generated (anti-K267me) and tested by dot blot assay against unmodified and chemically monomethylated K267 containing peptides (263–271) (Supplementary Figure S2a). By ESI-LC-MS analysis, overexpressed HA-MEF2D was immunoprecipitated with anti-K267me, pulling doen mono- and dimethylated MEF2D, demonstrating that anti-K267me recognizes the mono- and dimethylated forms of MEF2D (Figure 1a). Wild-type (WT) HA-MEF2D was detected using anti-K267me, but the K267R mutant was not (Figure 1b).

The detection of methylated MEF2D by anti-K267me was blocked with a chemically methylated K267-containing peptide (Figure 1c). Endogenous MEF2D immunoprecipitated with anti-K267me, demonstrating that MEF2D in proliferating C2C12 cells is methylated at K267 (Figure 1d). Furthermore, the proportion of methylated MEF2D in proliferating C2C12 cells was determined by immunodepletion assay (Figure 1e). On immunoprecipitation with anti-K267me, much of the MEF2D was depleted, suggesting the significance of this modification.

MEF2D methylation was also observed in DO11.10 T cells and mouse embryonic stem cells (E14) (Supplementary Figure S2b and d). Notably, K267 was highly conserved between MEF2 isoforms and species, suggesting critical functions for this residue and its modification (Supplementary Figure S2e and f). Moreover, lysine methylation at K267 in other MEF2 isoforms implicates a significant function of this modification in the regulation of MEF2 activity (Supplementary Figure S2g). The methylation-deficient mutants MEF2A and MEF2C could not be immunoprecipitated with anti-K267me (Supplementary Figure S2h and i).

To examine the significance of MEF2methylation, methylation levels of MEF2 during C2C12 cell differentiation were measured by western blot. MEF2 methylation levels declined during myogenesis, whereas total MEF2D levels increased (Figure 1f). Similarly, methyl-MEF2D levels decreased on ionomycin treatment or random differentiation in DO11.10 T cells and E14 cells (Supplementary Figure S2c and d). Alterations in MEF2D methylation level due to environmental changes implicate methylation as a regulatory mechanism of MEF2D.

### MEF2D is methylated by G9a

To identify the lysine methyltransferase (PKMT) that methylates MEF2D at K267, we screened PKMTs that are differentially expressed during C2C12 cell differentiation, during which MEF2D transcriptional activity is dynamically regulated. Using previous microarray data, we selected PKMTs that were differentially expressed during myogenesis. mRNA levels of these PKMTs during C2C12 cell differentiation were confirmed (32). C2C12 cells were cultured in differentiation medium (DM) for 2 or 4 days (DM2 or DM4) and harvested for RNA extraction.

Of the PKMTs, *Ezh2*, *G9a* and *Suv39h1* mRNA levels changed dramatically and were selected as candidates for MEF2D methylation (Supplementary Figure S3a). Suv39h1 as a methyltransferase that mediates trimethylation (2), but MEF2D is mono- and dimethylated (Figure 1a and Supplementary Figure S1); thus, Suv39h1 is unlikely the enzyme that methylates MEF2D.

**Figure 2.**
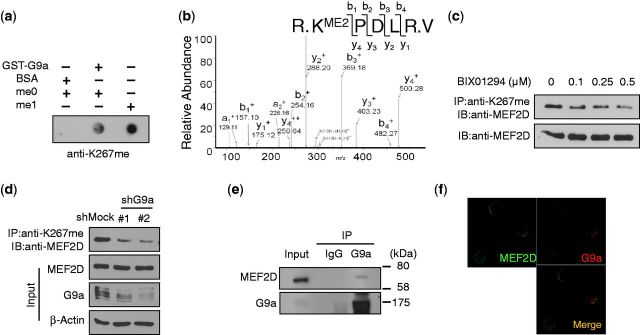
G9a methylates MEF2D at K267 through their interaction. (**a**) *In vitro* methylation of MEF2D peptide (263–271) (me0) by G9a was analyzed by dot blot assay. Chemically methylated MEF2D peptide (me1) was used as a positive control for antibody detection. (**b**) *In vitro* methylation of bacterially purified full-length His-MEF2D with G9a was analyzed by ESI-LC-MS. (**c**) C2C12 cells were treated with BIX01294 at the indicated concentrations. MEF2D and its methylation levels were analyzed by immunoprecipitation. (**d**) MEF2D methylation in C2C12 cells infected with shMock or shG9a was analyzed by immunoprecipitation. (**e**) Whole-cell lysates of C2C12 cells in GM were immunoprecipitated with anti-G9a or rabbit normal IgG. (**f**) Colocalization of G9a and MEF2D in proliferating C2C12 cells was analyzed by immunostaining.

We examined the remaining candidates, Ezh2 and G9a. Ezh2 and G9a protein levels decreased during differentiation of C2C12 cells (Supplementary Figure S3b). To determine whether Ezh2 or G9a methylated MEF2D at K267, we performed an *in vitro* methylation assay, in which MEF2D peptide (263–271) was the substrate. Methylation of MEF2D peptide reacted with Ezh2 was not detected by dot blot assay (Supplementary Figure S3c), but G9a methylated unmodified peptide (K267me0) (Figure 2a). Methylation of MEF2D peptide at K267 by G9a was also detected by extracted ion chromatography (Supplementary Figure S3d).

To validate the enzymatic activity of G9a on MEF2D, full-length MEF2D protein was bacterially purified and used as the substrate in an *in vitro* methylation assay. By ESI-LC-MS of MEF2D that was incubated with G9a, MEF2D was dimethylated by G9a *in vitro* (Figure 2b). MEF2D methylation by G9a *in vitro* was confirmed by immunoprecipitation and western blot with anti-K267me (Supplementary Figure S3e).

To determine whether G9a methylated MEF2D K267 in cells, HA-MEF2D (WT) was transiently expressed in HEK293 cells with or without the G9a-specific inhibitor BIX01294 (33). Methylation of HA-MEF2D declined on inhibition of G9a activity by BIX01294 (Supplementary Figure S4a). Moreover, by western blot, endogenous methylation of MEF2D in C2C12 cells fell in a BIX01294 concentration-dependent manner (Figure 2c and Supplementary Figure S4b). By immunostaining, we noted decreased methylation of MEF2D on treatment with BIX01294 (Supplementary Figure S4c).

To confirm the methylation of MEF2D by G9a, G9a was knocked down with lentiviral shRNA. On downregulation of G9a, methylation levels fell (Figure 2d). Thus, MEF2D is a substrate of G9a, which methylates it at K267.

### MEF2D interacts with G9a

To confirm that MEF2D is a substrate of G9a, the interaction between MEF2D and G9a was observed by His pulldown assay with overexpressed Flag-G9a (Supplementary Figure S5a). MEF2 family members share a highly conserved N-terminal domain, the MADS/MEF domain, which is important for DNA binding and protein–protein interactions (8). The N-terminus of MEF2D is alternatively spliced during muscle differentiation. A ubiquitously expressed isoform, MEF2Dα1, binds to corepressors and a muscle-specific isoform, MEF2Dα2, interacts with Ash2L (34).Thus, we examined whether the binding domain of MEF2Dα1 interacted with G9a.

Truncated mutants of MEF2D—1–270 amino acids (N270) and 1–130 amino acids (N130)—and G9a were overexpressed in HEK293 cells and subjected to coimmunoprecipitation assay. The interaction between the MEF2D MADS/MEF2 domain (N130) and G9a suggested that G9a binds to all MEF2 family members (Supplementary Figure S5b). Whereas 464–1001 amino acids (464C) and 685–1001 amino acids (685C) truncated mutants of G9a interacted with MEF2D, truncated Flag-G9a, 936–1001 amino acids (936C) mutant was unable to bind, indicating that the ankyrin repeat domain (amino acids 685–936) of G9a is required for the interaction (Supplementary Figure S5c).

The endogenous interaction between G9a and MEF2D was verified in proliferating C2C12 cells (Figure 2e). G9a and MEF2D colocalized in the nucleus of C2C12 cells by immunostaining (Figure 2f).

### MEF2D is demethylated by LSD1

**Figure 3.**
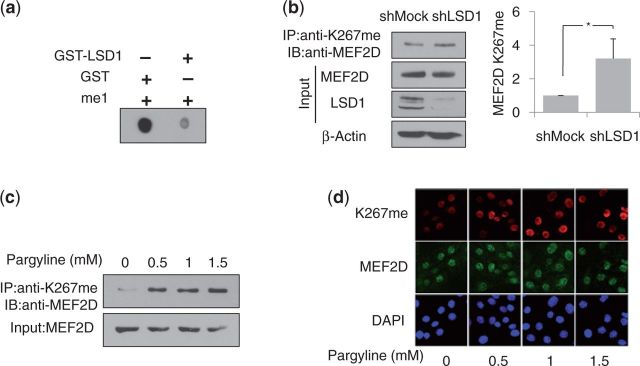
LSD1 demethylates MEF2D. (**a**) *In vitro* demethylation assay of monomethylated MEF2D peptide (263–271) (me1) by LSD1 was analyzed by dot blot assay. (**b**) MEF2D methylation in C2C12 cells infected with shMock or shLSD1 was analyzed by immunoprecipitation (left panel). Quantification of MEF2D methylation was depicted after normalization to total MEF2D levels (*n* = 3). (**c** and **d**) C2C12 cells were treated with pargyline at the indicated concentrations. MEF2D and its methylation were analyzed by immunoprecipitation (c) and immunostaining (d).

We have reported that LSD1 activates MEF2 during skeletal muscle differentiation (17). Thus, we hypothesized that LSD1 increases MEF2 transcriptional activity by demethylating K267, counteracting the function of G9a. By *in vitro* demethylase assay using a chemically modified K267-containing peptide, LSD1 demethylated K267 (Figure 3a), which was confirmed by the increase in K267 methylation in LSD1-knockdown C2C12 cells (Figure 3b and Supplementary Figure S6). Furthermore, MEF2 methylation rose on treatment with an LSD1 inhibitor, pargyline, in a concentration-dependent manner by immunoprecipitation and immunostaining (Figure 3c and d). This demethylase activity against MEF2D indicates that LSD1 regulates MEF2D transcriptional activity by modulating a histone modification, as reported (17), and by directly regulating lysine methylation of MEF2D.

### G9a inhibits MEF2D transcriptional activity by regulating its recruitment to chromatin

Next, we examined whether G9a modulates MEF2D transcriptional activity by methylating it. The luciferase gene, controlled by a promoter with an artificial MEF2 element, was transfected into HEK293 cells with or without MEF2D, alone and withG9a. On increased expression of G9a, MEF2D transcriptional activity on the MEF2 element-containing and *myogenin* promoters declined (Supplementary Figure S7a and b).

**Figure 4.**
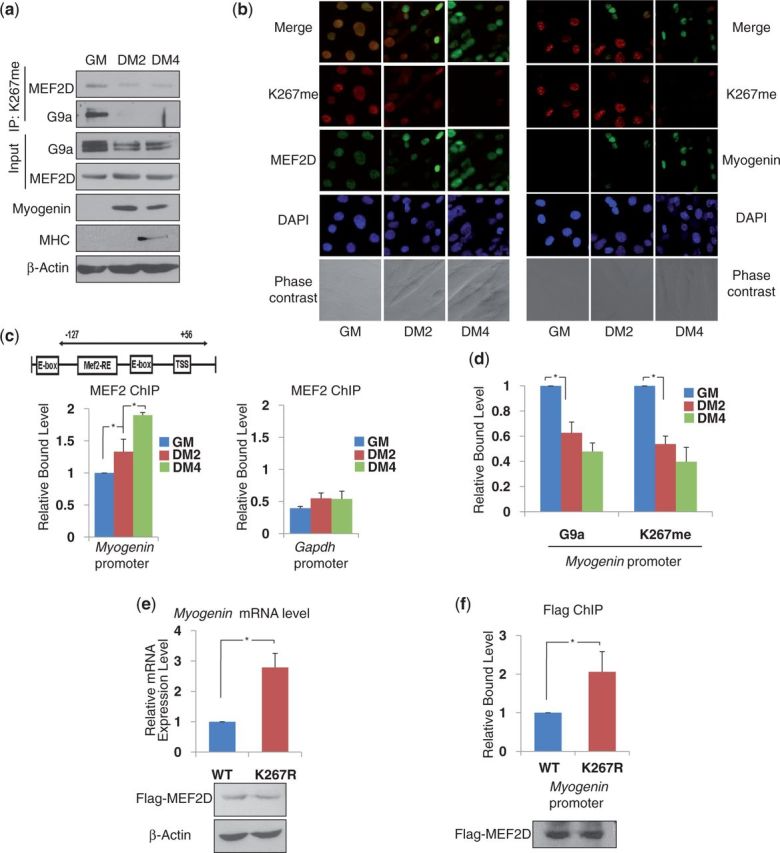
MEF2D transcription activity is repressed by G9a. (**a**) C2C12 cells were differentiated for up to 4 days. Whole-cell lysates of C2C12 cells in GM, DM2 and DM4 were immunoprecipitated and immunoblotted with the indicated antibodies. (**b**) Differentiated C2C12 cells were analyzed by immunostaining with the indicated antibodies. (**c**) ChIP assays were performed with anti-MEF2 using differentiated C2C12 cells. Immunoprecipitated DNA fragments were analyzed for *the myogenin* or *gapdh* promoter and expressed, relative to the level of *myogenin* promoter bound in GM. (**d**) ChIP assays were performed with the indicated antibodies using differentiated C2C12 cells. Immunoprecipitated DNA fragments were analyzed for *the myogenin* promoter and expressed, relative to the bound level in GM. (**e**) C2C12 cells overexpressing Flag-MEF2D (WT) or Flag-MEF2D (K267R) were harvested for RNA preparation. *Myogenin* mRNA level was analyzed by qRT–PCR. (**f**) C2C12 cells overexpressing Flag-MEF2D (WT) or (K267R) were harvested for ChIP assay. Immunoprecipitated DNA fragments were analyzed for *the myogenin* promoter and expressed, relative to the level of MEF2D bound (WT). *P < 0.05.

To determine the significance of the regulation of MEF2D activity by G9a and its methylation, methylation levels and the expression of MEF2D target genes were monitored during C2C12 cell differentiation, during which MEF2D transcriptional activity is elevated. Increased transcriptional activity of MEF2D was reflected by the upregulation of MEF2D target genes and myogenic markers. Consequently, with enhanced MEF2 activity, MEF2D K267 methylation decreased, as did its interaction with G9a (Figure 4a and Supplementary Figure S7c). By immunostaining, we noted the inverse between MEF2D activity and methylation (Figure 4b).

Moreover, on Day 2 of differentiation, cells with methylated MEF2D did not express myogenin, whereas myogenin-expressing cells showed no MEF2D methylation (Figure 4b). These results indicate that G9a inhibits MEF2D transcriptional function through K267 methylation.

Next, we investigated whether G9a occupied MEF2D binding sites and regulated MEF2D activity on target promoters. The *myogenin* promoter, which contains an MEF2-binding site, was activated by MEF2D during myogenesis (Figure 4c). MEF2 bound to the promoter in C2C12 cells in growth medium (GM); this binding increased in differentiating C2C12 cells (Figure 4c). By ChIP assay, G9a bound to the *myogenin* promoter in proliferating C2C12 cells but dissociated as C2C12 cells differentiated (Figure 4d).

Moreover, K267 methylation of MEF2D was observed in the *myogenin* promoter in undifferentiated C2C12 cells but disappeared as C2C12 cells differentiated, similar to the binding pattern of G9a (Figure 4d). Thus, G9a represses MEF2D activity by methylating it on target promoters, depending on the stage of differentiation. G9a and MEF2 were also detected on the MCK promoter, another MEF2 target gene (Supplementary Figure S8).

Next, the methylation-deficient mutant MEF2D (K267R) was overexpressed in C2C12 cells to assess its activity on target genes. The transcriptional activity of MEF2D (K267R) was derepressed, as evidenced by the increased expression of *myogenin* compared with C2C12 cells that overexpressed MEF2D (WT) (Figure 4e).

Because a lysine residue that corresponds to MEF2D K267 is a site of acetylation that affects DNA binding by MEF2C (22), we sought to determine the effects of methylation on the recruitment of MEF2D to chromatin. The DNA binding of Flag-MEF2D (WT) and Flag-MEF2D (K267R) in proliferating C2C12 cells was measured by ChIP assay—MEF2D (K267R) demonstrated enhanced DNA binding compared with MEF2D (WT) (Figure 4f). These results indicate that MEF2D methylation by G9a regulates its recruitment to chromatin and transcriptional activity.

### Knockdown of G9a enhances MEF2D-dependent transcription

**Figure 5.**
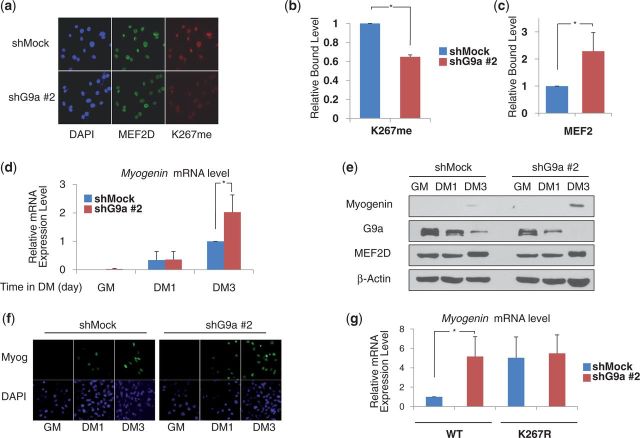
Knockdown of G9a decreases MEF2D methylation and enhances its activity. (**a**) MEF2D methylation in C2C12 cells infected with shMock or shG9a was analyzed by immunostaining with the indicated antibodies. (**b** and **c**) ChIP assays were performed with anti-K267me (**b**) or anti-MEF2 (**c**) using proliferating C2C12 cells. Immunoprecipitated DNA fragments were analyzed for *myogenin* promoter and expressed, relative to the bound level in shMock. *P < 0.05. (**d**) shMock- or shG9a-infected C2C12 cells were differentiated and harvested at the times indicated. *Myogenin* mRNA levels were analyzed by qRT–PCR. *P < 0.05. (**e** and **f**) C2C12 cells were differentiated as in (d) and analyzed by immunoblotting (**e**) or immunostaining (**f**) with the indicated antibodies. (**g**) C2C12 cells infected with shMock or shG9a were infected with pMIG-MEF2D (WT) or MEF2D (K267R). *Myogenin* mRNA level was analyzed by qRT–PCR. *P < 0.05.

To determine the effects of the dysregulation of G9a and MEF2D methylation, G9a was knocked down in C2C12 cells using shRNA-expressing lentiviral vectors. On downregulation of G9a, MEF2D methylation decreased by immunostaining (Figure 5a). Also, knockdown of G9a lowered MEF2D methylation levels on the *myogenin* promoter (Figure 5b). Consistent with previous results, the level of MEF2 that bound to the *myogenin* promoter rose in G9a knockdown C2C12 cells (Figure 5c). Consequently, the mRNA of MEF2D target genes and the myogenic markers *myogenin*, *MCK,* and *MHC* was upregulated (Figure 5d and Supplementary Figure S9a). Moreover, by western blot, Myogenin levels increased (Figure 5e). Expression of myogenin and differentiation were stimulated in C2C12 cells with lower levels of G9a (Figure 4f and Supplementary Figure S9b).

To confirm the effect of MEF2D methylation with regard to the enhanced transcriptional activity of MEF2D by G9a knockdown, MEF2D (WT) or MEF2D (K267R) was overexpressed in G9a knockdown and control C2C12 cells. Proliferating cells were harvested and *myogenin* mRNA was analyzed. Overexpressed MEF2D (WT) had higher transcriptional activity in G9a knockdown cells versus control cells (Figure 5g). Moreover, *myogenin* mRNA levels in MEF2D (K267R)-overexpressing control cells were comparable with those in G9a knockdown cells that overexpressed MEF2D (WT) and MEF2D (K267R) (Figure 5g). Furthermore, the transcriptional activities of MEF2D (K267R) were similar, regardless of the presence of G9a (Figure 5g). These data indicate that C2C12 cells in which G9a has been knocked down show enhanced transcriptional activity of MEF2D due to inadequate methylation at K267. Thus, our findings confirm that G9a knockdown significantly decreases MEF2 methylation, inducing muscle differentiation.

### Overexpression of G9a attenuates MEF2D transcriptional activity

**Figure 6.**
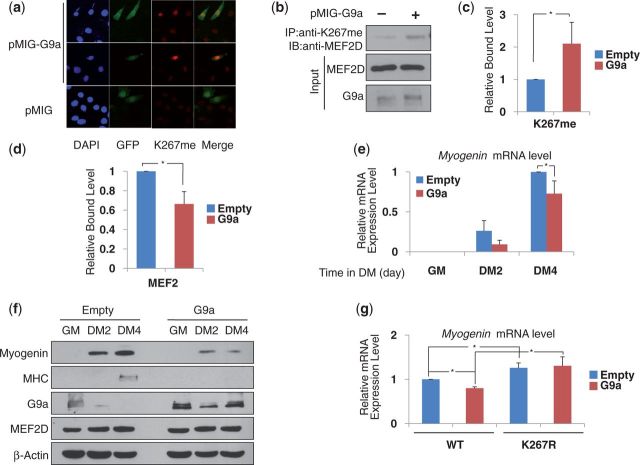
Overexpression of G9a increases MEF2D methylation and represses its activity. (**a** and **b**) Retrovirus expressing G9a (pMIG-G9a) was used to infect C2C12 cells. Methylation of MEF2D K267 was analyzed by immunostaining (**a**) and immunoprecipitation (**b**). (**c**) ChIP assays were performed with anti-K267me using proliferating C2C12 cells. Immunoprecipitated DNA fragments were analyzed for *myogenin* promoter and expressed, relative to the bound level in GM. (**d**) C2C12 cells stably overexpressing G9a were differentiated and harvested for ChIP assay with anti-MEF2. (**e**) C2C12 cells stably overexpressing G9a were differentiated and harvested at the times indicated. *Myogenin* and *MHC* mRNA levels were analyzed by qRT–PCR. (**f**) C2C12 cells were differentiated as in (**e**) and analyzed by immunoblotting with the indicated antibodies. (**g**) C2C12 cells stably overexpressing G9a were transfected with Flag-MEF2D (WT) or Flag-MEF2D (K267R) and differentiated for 24 h. *Myogenin* mRNA level was analyzed by qRT–PCR. *P < 0.05.

To validate the function of G9a with regard to MEF2D transcriptional activity, the effects of G9a overexpression on MEF2D targets were determined during skeletal muscle cell differentiation. G9a was overexpressed in C2C12 cells by infecting them with a retrovirus that coexpressed GFP and G9a. By immunostaining, MEF2D methylation was induced in cells that were infected with G9a-expressing retrovirus (Figure 6a). MEF2D methylation also rose with exogenous expression of G9a by western blot (Figure 6b). Moreover, overexpression of G9a increased MEF2D methylation in the *myogenin* promoter (Figure 6c).

The enhanced methylation of MEF2D by G9a overexpression resulted in a decrease in promoter-bound MEF2 in differentiated C2C12 cells (Figure 6d), consequently repressing MEF2-dependent transcription (Figure 6e). The mRNA and protein levels of MEF2 target genes and myogenic markers were downregulated in G9a-overexpressing C2C12 cells (Figure 6e and f). The reduction in myogenin was also observed by immunostaining (Supplementary Figure S10a). Furthermore, C2C12 cells that experienced increased MEF2D methylation due to forced expression of G9a underwent impaired differentiation, as evidenced by immunostaining with anti-MHC (Supplementary Figure S10b).

To determine the significance of MEF2 methylation in G9a-mediated inhibition of myogenesis, MEF2D (WT) or MEF2D (K267R) was overexpressed in C2C12 cells that stably expressed empty vector (Empty) or G9a (Supplementary Figure S11), and *myogenin* mRNA was measured after 24 h of differentiation. Activity of transiently overexpressed MEF2D (WT) was inhibited by G9a, as reflected by the decrease in *myogenin* in G9a-expressing C2C12 cells compared with empty vector-expressing C2C12 cells (Figure 6g). However, MEF2D (K267R) activity was unaffected by G9a, as shown by the comparable *myogenin* levels between C2C12 cells that expressed empty vector and G9a (Figure 6g). These data demonstrate that in G9a-overexpressing C2C12 cells, MEF2D-dependent transcription and differentiation are inhibited by MEF2 methylation on K267.

## DISCUSSION

In this study, we determined that MEF2 activity is regulated by reversible lysine methylation, a novel posttranslational modification and newly identified G9a as a corepressor that methylates and regulates MEF2. The methylation of lysine in the transactivation domain, governed by G9a and LSD1, represses MEF2D transcriptional activity, effecting the downregulation of target genes. Our previous and current findings demonstrate that dysregulation of MEF2D methylation due to aberrant expression of G9a and LSD1 impedes and enhances myogenesis, respectively (17).

Transcription factors that lie upstream in transcription cascades are regulated by such mechanisms as posttranslational modifications to obtain strict control over a broad range of target genes. In *Drosophila*, in which a single dMEF2 regulates muscle development, MEF2 levels differentially regulate diverse patterns of target gene expression (10,35). In vertebrate, four isoforms of MEF2 exist and are heavily modified by various kinases and acetyltransferases to regulate its activity over time and by location (9). In particular, the lysine residue of MEF2C that corresponds to residue 267of MEF2D is acetylated by p300 (22), increasing its DNA binding and transcriptional activity (22), implicating the interactive regulation of MEF2 activity by methylation and acetylation. In myoblasts, MEF2 activity remains repressed by methylation, and MEF2 is demethylated on the initiation of differentiation cues. To fully activate MEF2, p300 is recruited and acetylates MEF2 in myogenesis, constituting a methylation–acetylation switch (Supplementary Figure S12).

In addition, methylation might suppress the phosphorylation of nearby sites that are targeted by p38α. The region that encompasses lysine 267 is called the D-domain—i.e., the p38α docking domain (20). Thus, G9a-dependent methylation of lysine 267might block the p38α–MEF2 interaction in myoblasts to maintain MEF2 activity at basal levels. Thus, it is possible that lysine methylation cooperates with other posttranslational modifications in determining the promoter- and differentiation stage-specific transcriptional activity in a complex gene activation program during myogenesis.

Among the corepressors that are recruited to MEF2 target promoters, Cabin1 has been reported to coordinate histone-modifying enzymes to regulate transcription of MEF2 target genes (12,14). G9a, a novel corepressor of MEF2 that we have identified, interacts with Cabin1 (36). Thus, we speculate that Cabin1 forms a complex with G9a, in addition to Suv39h1 and HDACs, to modulate MEF2 activity. Sharp-1, a repressor of muscle differentiation (37), is another possible cofactor that mediates the repression of MyoD by G9a. Sharp-1 augments MyoD methylation by G9a and suppresses MyoD activity (38). Thus, Sharp-1 might function as an adaptor protein in G9a-dependent MEF2 methylation and repression.

Among the nonhistone proteins that are targeted by G9a, MyoD is a substrate of G9a (2,39). MyoD is a myogenic regulator that shares target genes with MEF2 to synergistically induce skeletal muscle differentiation (18). Binding elements of MyoD and MEF2 on common target promoters are positioned closely, allowing them to bind as a dimer (18). It is likely that MyoD and MEF2 share cofactors and are regulated similarly, because LSD1 also interacts and both MyoD and MEF2 to enhance myogenic differentiation (17). Thus, having demonstrated the mechanism by which G9a- and LSD1-mediated methylation regulates MEF2, our data support that G9a and LSD1 are the critical epigenetic regulators that govern two major classes of myogenic transcription factors and control skeletal muscle differentiation precisely.

Our findings indicate that G9a and LSD1 regulate MEF2D transcriptional activity through methylation of lysine 267. In myoblast cells with basal MEF2 activity, G9a is expressed and bound to MEF2 target promoters. MEF2 methylation is high, and its target genes are undetected at the mRNA and protein levels. During differentiation, G9a mRNA and protein levels decrease. Consequently, the amount of G9a that binds to MEF2 target promoters declines. Moreover, the interaction between G9a and MEF2D decreases, while MEF2D methylation diminishes. This study implicates a mechanism in which G9a and LSD1 regulate MEF2D activity over time and by location by a novel posttranslational modification, lysine methylation.

## SUPPLEMENTARY DATA

Supplementary Data are available at NAR Online

## FUNDING

Funding for open access charge: National Research Foundation of Korea (NRF); Korean government (MSIP) [National Creative Research Laboratory Program (2012R1A3A2048767 to H.-D.Y.]; Mid-Career Research Program [NRF-2007-0056786 & NRF-2010-0007643 to H.-D.Y., and ROA-2008-0060084 to E.-J.C.]; Basic Science Research Program [2010-0028646 to E.-J.C.]; World Class University Program of the MEST and the NRF [R31-2008-000-10103-0 to H.-D.Y.]; Supported by a Seoul Science Fellowship (to J.C.).

*Conflict of interest statement*. None declared.

## Supplementary Material

Supplementary Data
