# Supplementary material for: Modulation of lysine methylation in myocyte enhancer factor 2 during skeletal muscle cell differentiation
Source: Nucleic Acids Res. 2013 Sep 27;42(1):224–34. doi: 10.1093/nar/gkt873 (PMC3874188; doi:10.1093/nar/gkt873)
Supplement: Supplementary Data [file supp_42_1_224__index.html]

Modulation of lysine methylation in myocyte enhancer factor 2 during skeletal muscle cell differentiation — Modulation of lysine methylation in myocyte enhancer factor 2 during skeletal muscle cell differentiation — Supplementary Data 

# Modulation of lysine methylation in myocyte enhancer factor 2 during skeletal muscle cell differentiation

## Supplementary Data

files

**Files in this Data Supplement:**

- Supplementary Data - pptx file
