# Supplementary material for: Modulation of lysine methylation in myocyte enhancer factor 2 during skeletal muscle cell differentiation
Source: Nucleic Acids Res. 2013 Sep 27;42(1):224–34. doi: 10.1093/nar/gkt873 (PMC3874188; doi:10.1093/nar/gkt873)
Supplement: Supplementary Data [file supp_gkt873_nar-01634-x-2013-File003.pptx]

## Slide 1
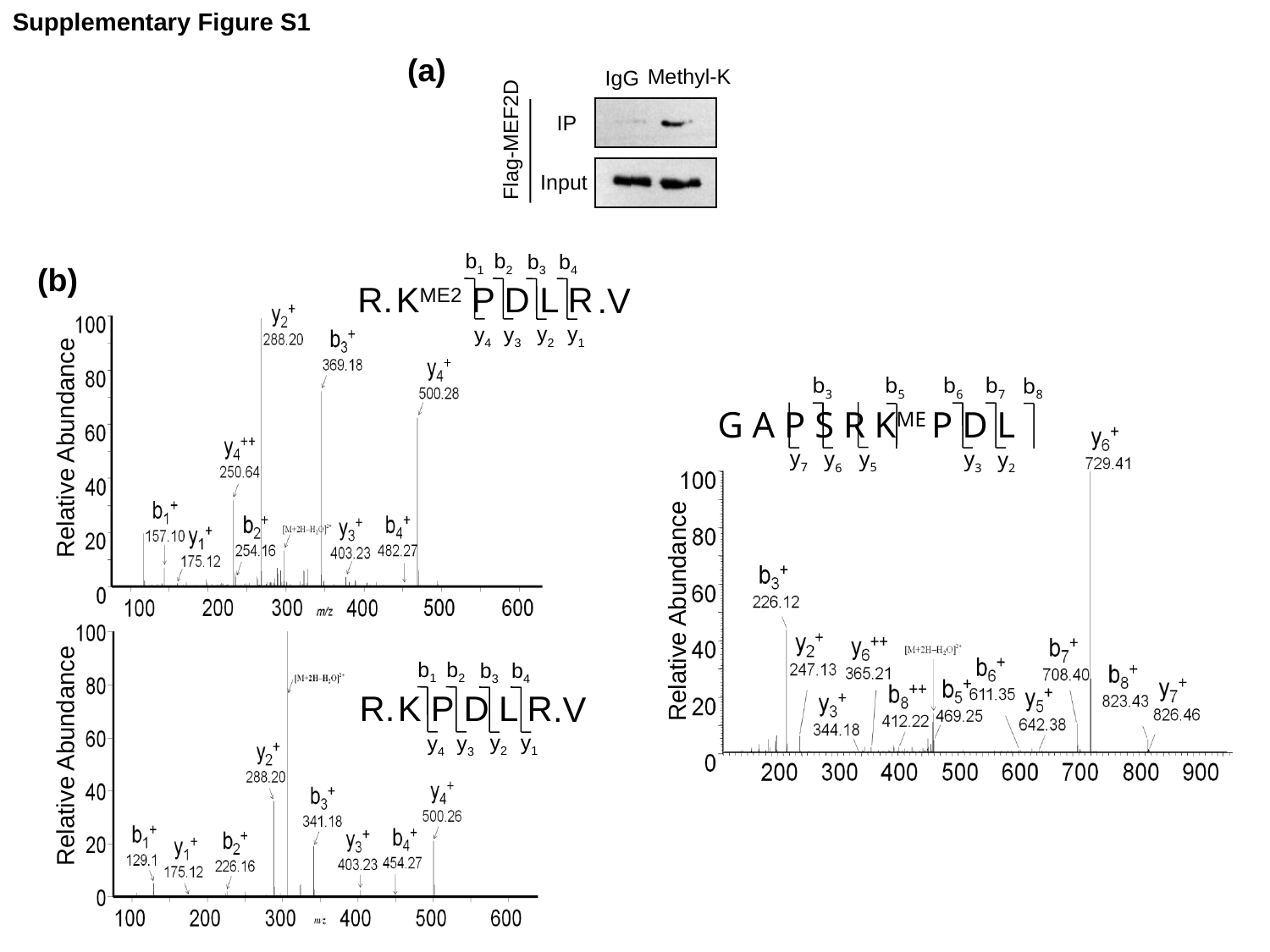

Supplementary Figure S1
(a)
Methyl-K
IgG
IP
Flag-MEF2D
Input
b1
b2
b3
b4
R.
KME2 P D L R
.V
y2
y1
y4
y3
(b)
b3
b5
b6
b7
b8
G A P S R KME P D L
y7
y5
y6
y3
y2
Relative Abundance
Relative Abundance
b1
b2
b3
b4
R.
K P D L R
.V
y2
y1
y4
y3
Relative Abundance

## Slide 2
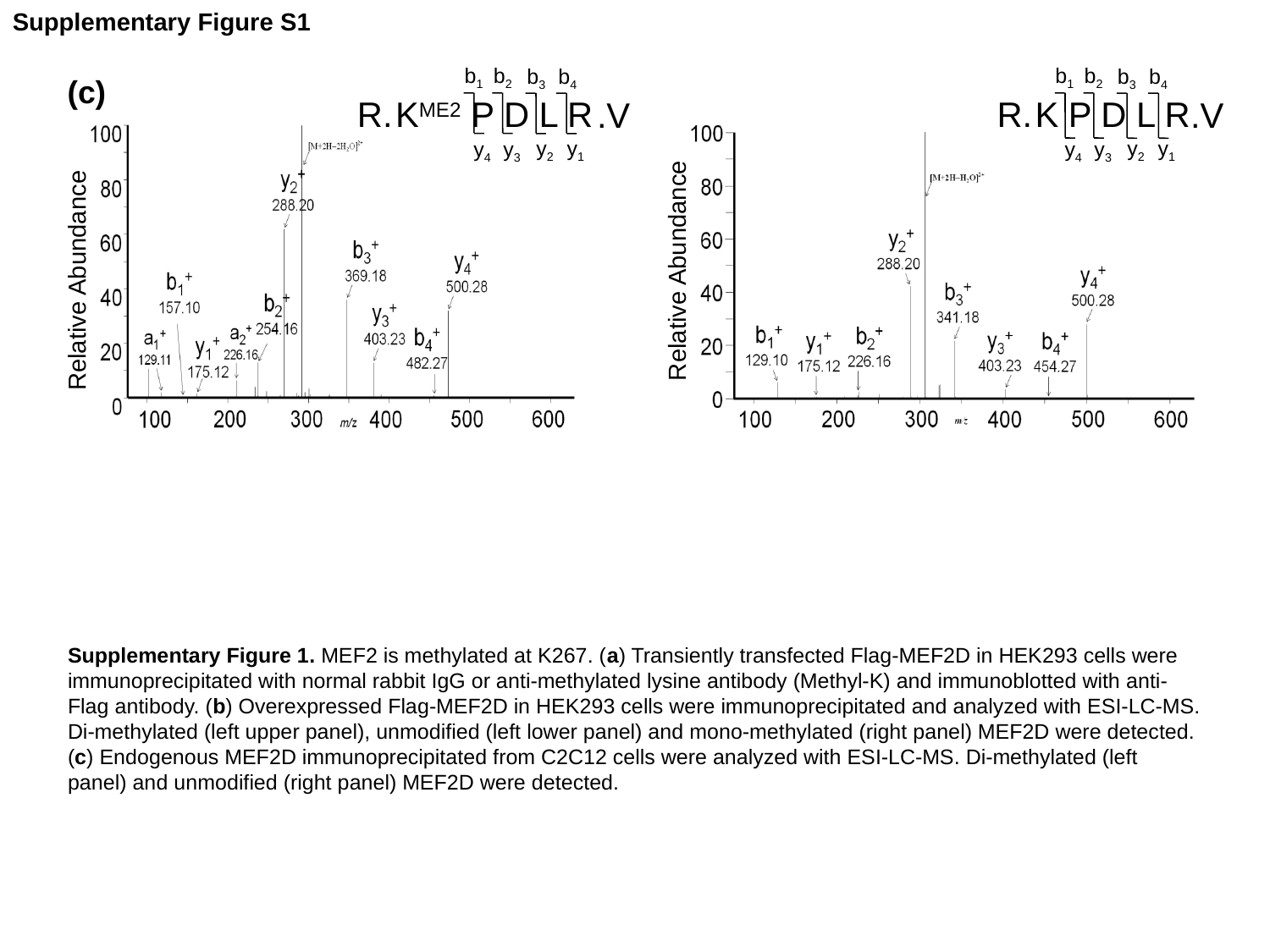

Supplementary Figure S1
b1
b2
b3
b4
R.
KME2 P D L R
.V
y2
y1
y4
y3
b1
b2
b3
b4
R.
K P D L R
.V
y2
y1
y4
y3
(c)
Relative Abundance
Relative Abundance
Supplementary Figure 1. MEF2 is methylated at K267. (a) Transiently transfected Flag-MEF2D in HEK293 cells were immunoprecipitated with normal rabbit IgG or anti-methylated lysine antibody (Methyl-K) and immunoblotted with anti-Flag antibody. (b) Overexpressed Flag-MEF2D in HEK293 cells were immunoprecipitated and analyzed with ESI-LC-MS. Di-methylated (left upper panel), unmodified (left lower panel) and mono-methylated (right panel) MEF2D were detected. (c) Endogenous MEF2D immunoprecipitated from C2C12 cells were analyzed with ESI-LC-MS. Di-methylated (left panel) and unmodified (right panel) MEF2D were detected.

## Slide 3
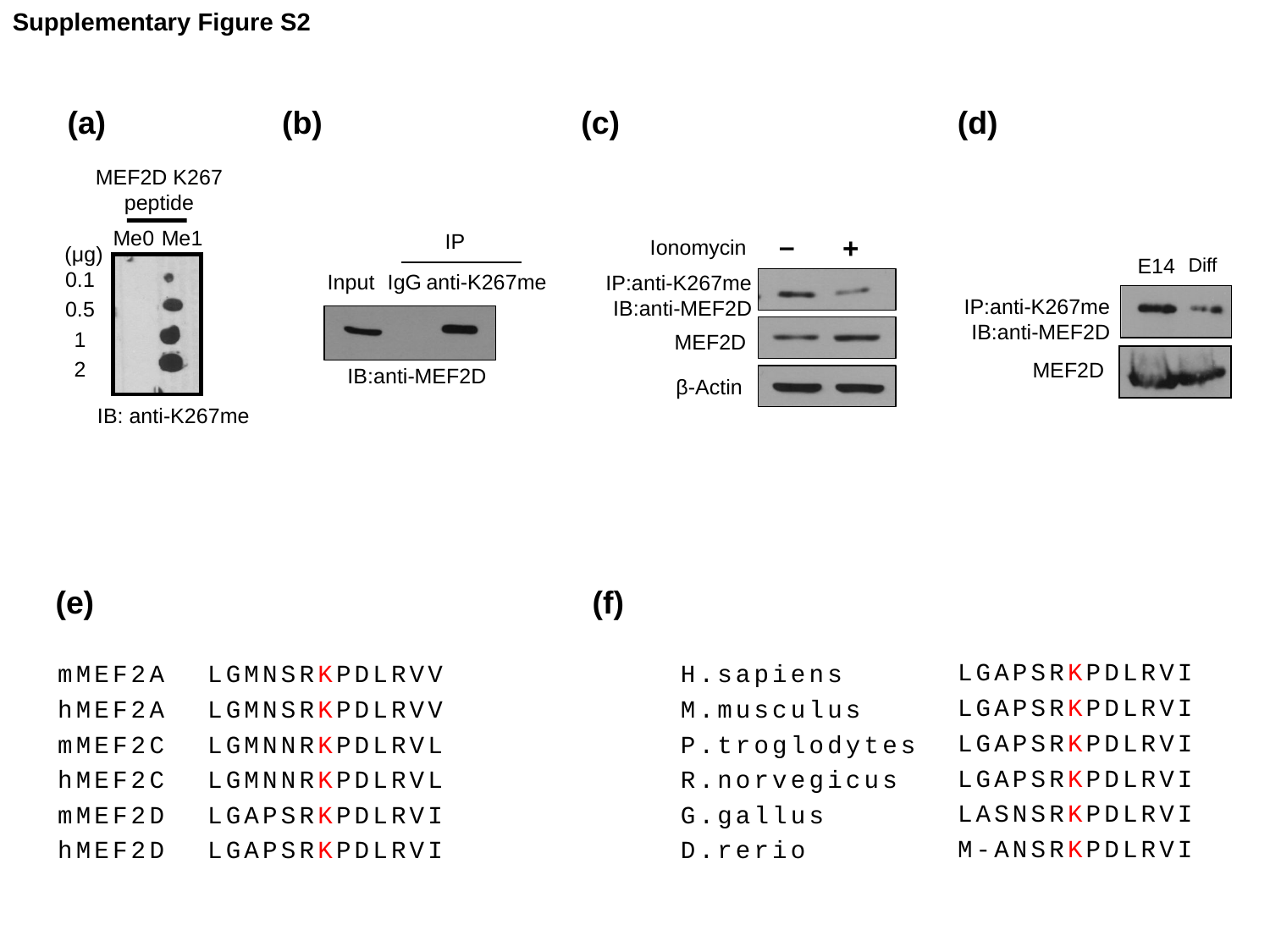

Supplementary Figure S2
(a)
(b)
(c)
(d)
MEF2D K267
peptide
Me0
Me1
IP
–
 +
Ionomycin
 (μg)
E14
Diff
0.1
IgG
anti-K267me
Input
IP:anti-K267me IB:anti-MEF2D
IP:anti-K267me IB:anti-MEF2D
0.5
1
MEF2D
2
MEF2D
IB:anti-MEF2D
β-Actin
IB: anti-K267me
(e)
(f)
mMEF2A
hMEF2A
mMEF2C
hMEF2C
mMEF2D
hMEF2D
LGMNSRKPDLRVV
LGMNSRKPDLRVV
LGMNNRKPDLRVL
LGMNNRKPDLRVL
LGAPSRKPDLRVI
LGAPSRKPDLRVI
H.sapiens
M.musculus
P.troglodytes
R.norvegicus
G.gallus
D.rerio
LGAPSRKPDLRVI
LGAPSRKPDLRVI
LGAPSRKPDLRVI
LGAPSRKPDLRVI
LASNSRKPDLRVI
M-ANSRKPDLRVI

## Slide 4
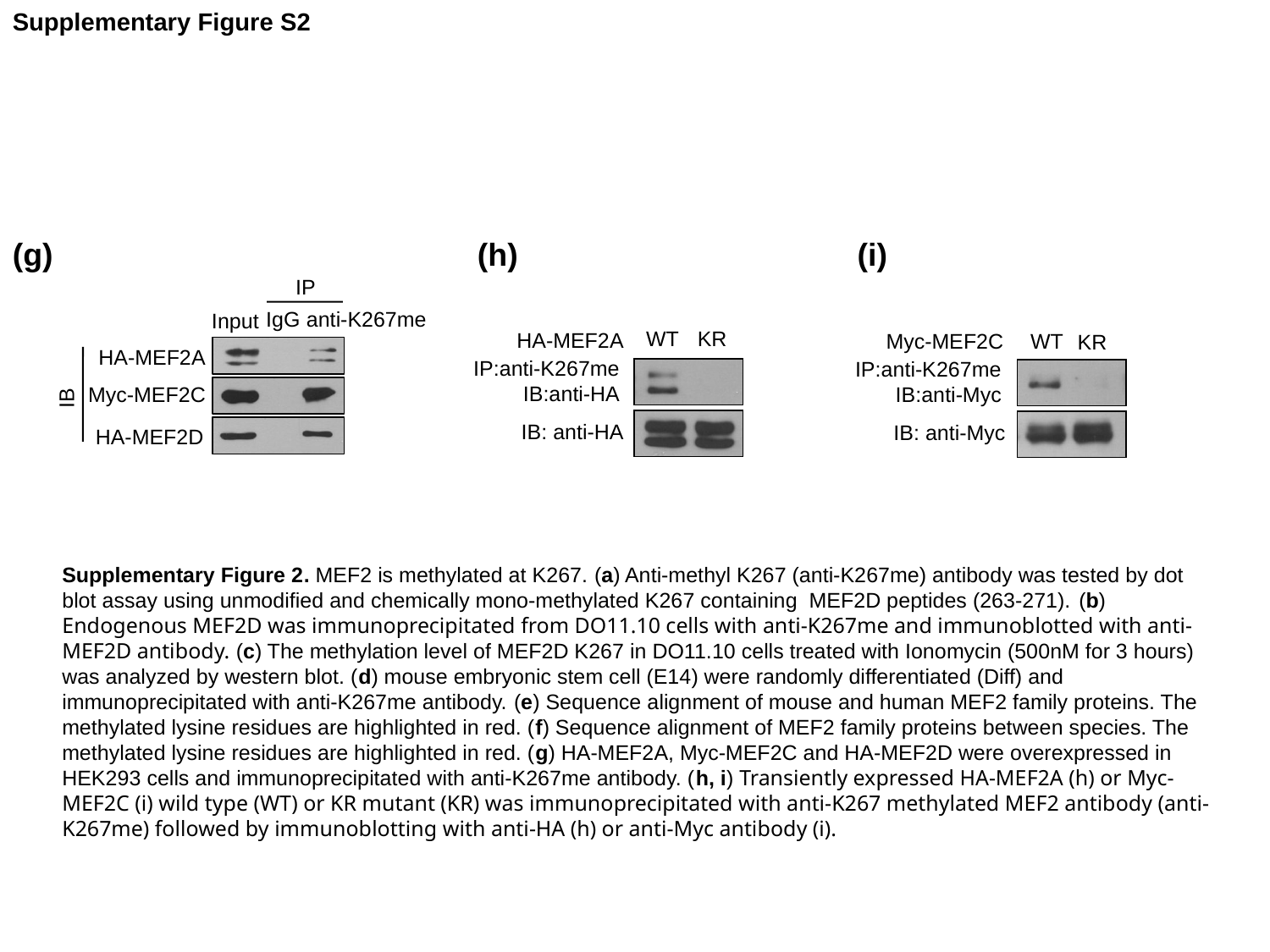

Supplementary Figure S2
(g)
(h)
(i)
IP
IgG
anti-K267me
Input
WT
KR
HA-MEF2A
Myc-MEF2C
WT
KR
HA-MEF2A
IP:anti-K267me IB:anti-HA
IP:anti-K267me IB:anti-Myc
Myc-MEF2C
IB
IB: anti-HA
IB: anti-Myc
HA-MEF2D
Supplementary Figure 2. MEF2 is methylated at K267. (a) Anti-methyl K267 (anti-K267me) antibody was tested by dot blot assay using unmodified and chemically mono-methylated K267 containing MEF2D peptides (263-271). (b) Endogenous MEF2D was immunoprecipitated from DO11.10 cells with anti-K267me and immunoblotted with anti-MEF2D antibody. (c) The methylation level of MEF2D K267 in DO11.10 cells treated with Ionomycin (500nM for 3 hours) was analyzed by western blot. (d) mouse embryonic stem cell (E14) were randomly differentiated (Diff) and immunoprecipitated with anti-K267me antibody. (e) Sequence alignment of mouse and human MEF2 family proteins. The methylated lysine residues are highlighted in red. (f) Sequence alignment of MEF2 family proteins between species. The methylated lysine residues are highlighted in red. (g) HA-MEF2A, Myc-MEF2C and HA-MEF2D were overexpressed in HEK293 cells and immunoprecipitated with anti-K267me antibody. (h, i) Transiently expressed HA-MEF2A (h) or Myc-MEF2C (i) wild type (WT) or KR mutant (KR) was immunoprecipitated with anti-K267 methylated MEF2 antibody (anti-K267me) followed by immunoblotting with anti-HA (h) or anti-Myc antibody (i).

## Slide 5
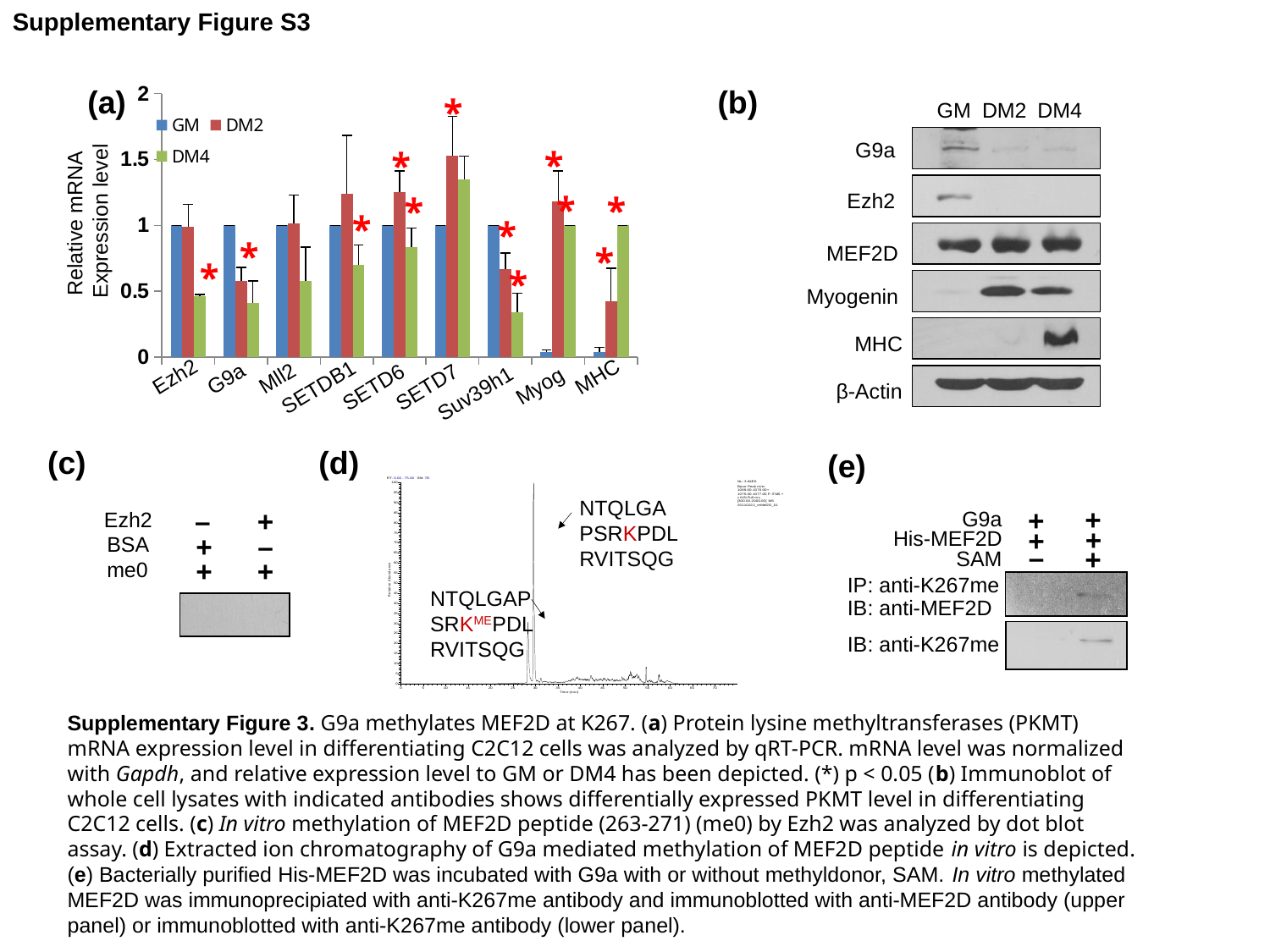

Supplementary Figure S3
(a)
(b)
### Chart
| Category | GM | DM2 | DM4 |
|---|---|---|---|*
*
*
*
*
*
*
*
*
*
*
*
DM4
GM
DM2
G9a
Ezh2
Relative mRNA
Expression level
MEF2D
Myogenin
MHC
Mll2
Ezh2
G9a
MHC
Myog
SETD6
SETD7
SETDB1
Suv39h1
β-Actin
(c)
(d)
(e)
NTQLGAPSRKPDLRVITSQG
NTQLGAPSRKMEPDLRVITSQG
 +
 +
–
+
G9a
Ezh2
 +
 +
His-MEF2D
+
–
BSA
–
 +
SAM
+
+
me0
IP: anti-K267me
IB: anti-MEF2D
IB: anti-K267me
Supplementary Figure 3. G9a methylates MEF2D at K267. (a) Protein lysine methyltransferases (PKMT) mRNA expression level in differentiating C2C12 cells was analyzed by qRT-PCR. mRNA level was normalized with Gapdh, and relative expression level to GM or DM4 has been depicted. (*) p < 0.05 (b) Immunoblot of whole cell lysates with indicated antibodies shows differentially expressed PKMT level in differentiating C2C12 cells. (c) In vitro methylation of MEF2D peptide (263-271) (me0) by Ezh2 was analyzed by dot blot assay. (d) Extracted ion chromatography of G9a mediated methylation of MEF2D peptide in vitro is depicted. (e) Bacterially purified His-MEF2D was incubated with G9a with or without methyldonor, SAM. In vitro methylated MEF2D was immunoprecipiated with anti-K267me antibody and immunoblotted with anti-MEF2D antibody (upper panel) or immunoblotted with anti-K267me antibody (lower panel).

## Slide 6
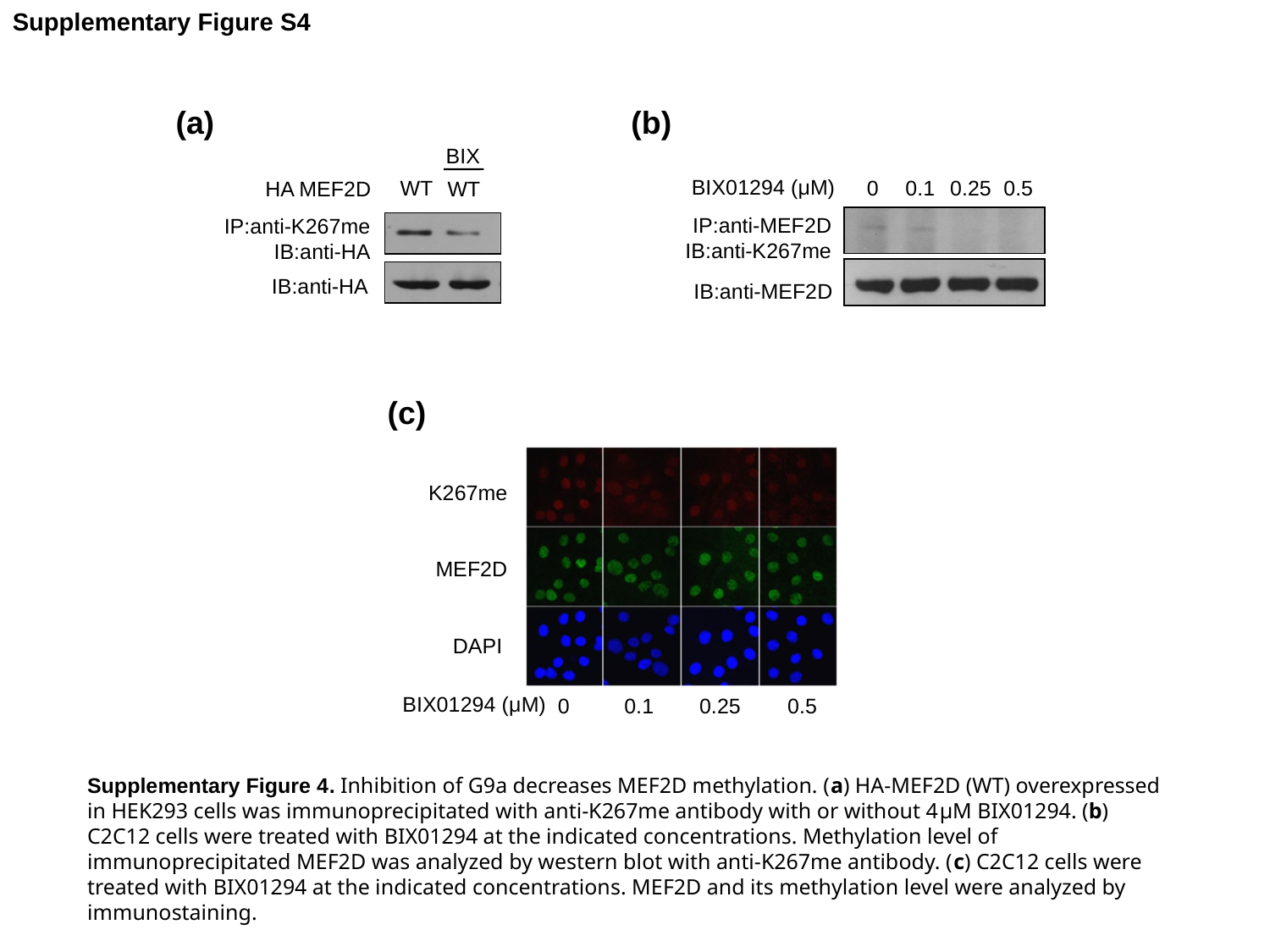

Supplementary Figure S4
(a)
(b)
BIX
BIX01294 (μM)
0
0.1
0.25
0.5
WT
HA MEF2D
WT
IP:anti-MEF2D IB:anti-K267me
IP:anti-K267me IB:anti-HA
IB:anti-HA
IB:anti-MEF2D
(c)
K267me
MEF2D
DAPI
BIX01294 (μM)
0
0.1
0.25
0.5
Supplementary Figure 4. Inhibition of G9a decreases MEF2D methylation. (a) HA-MEF2D (WT) overexpressed in HEK293 cells was immunoprecipitated with anti-K267me antibody with or without 4μM BIX01294. (b) C2C12 cells were treated with BIX01294 at the indicated concentrations. Methylation level of immunoprecipitated MEF2D was analyzed by western blot with anti-K267me antibody. (c) C2C12 cells were treated with BIX01294 at the indicated concentrations. MEF2D and its methylation level were analyzed by immunostaining.

## Slide 7
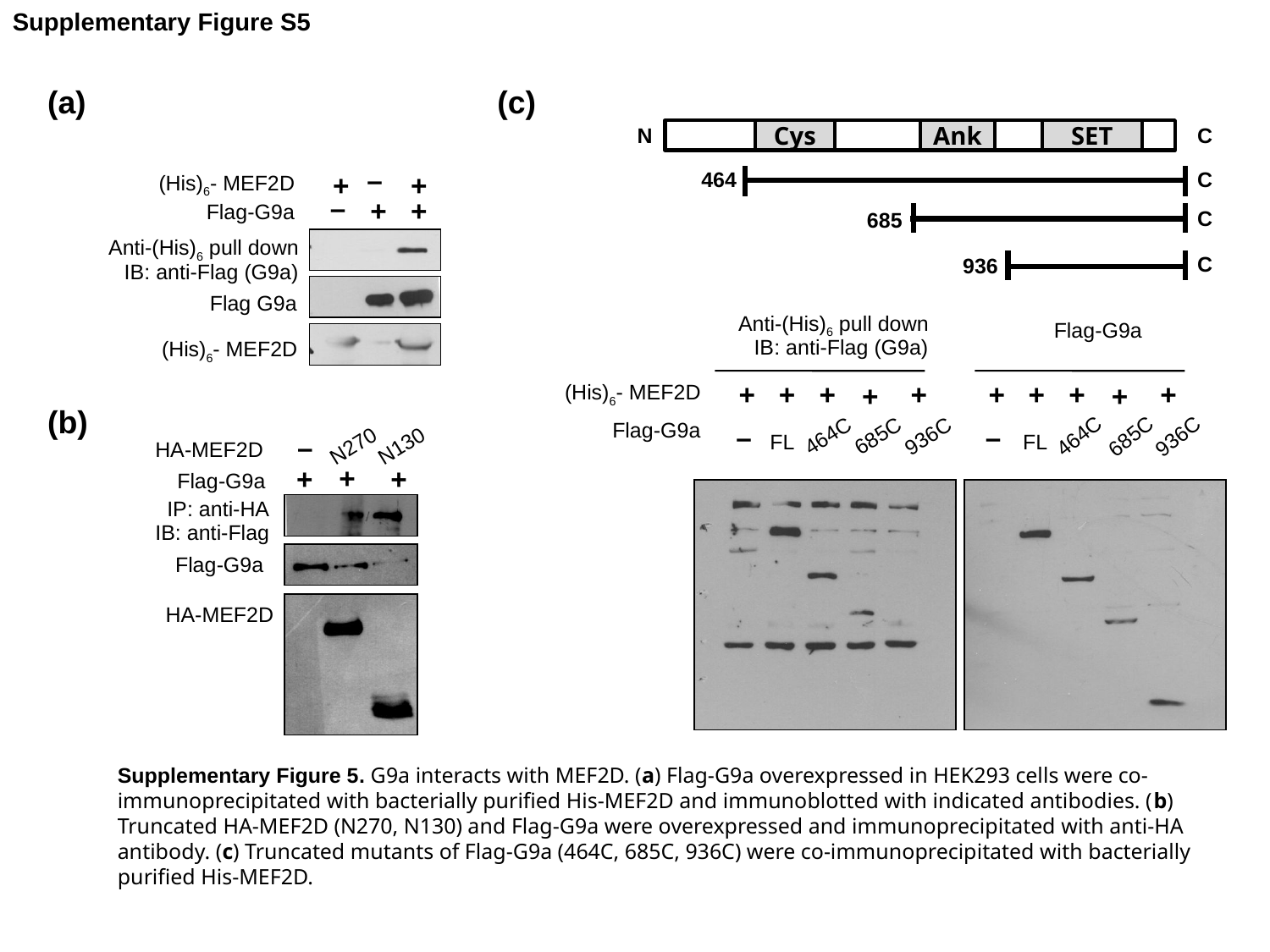

Supplementary Figure S5
(a)
(c)
N
C
Cys
Ank
SET
–
464
C
 +
 +
(His)6- MEF2D
–
 +
 +
Flag-G9a
C
685
Anti-(His)6 pull down
IB: anti-Flag (G9a)
C
936
Flag G9a
Anti-(His)6 pull down
IB: anti-Flag (G9a)
Flag-G9a
(His)6- MEF2D
 +
 +
 +
 +
 +
 +
 +
 +
 +
 +
(His)6- MEF2D
(b)
Flag-G9a
–
–
464C
464C
685C
685C
936C
936C
FL
FL
–
N130
N270
HA-MEF2D
 +
 +
 +
Flag-G9a
IP: anti-HA
IB: anti-Flag
Flag-G9a
HA-MEF2D
Supplementary Figure 5. G9a interacts with MEF2D. (a) Flag-G9a overexpressed in HEK293 cells were co-immunoprecipitated with bacterially purified His-MEF2D and immunoblotted with indicated antibodies. (b) Truncated HA-MEF2D (N270, N130) and Flag-G9a were overexpressed and immunoprecipitated with anti-HA antibody. (c) Truncated mutants of Flag-G9a (464C, 685C, 936C) were co-immunoprecipitated with bacterially purified His-MEF2D.

## Slide 8
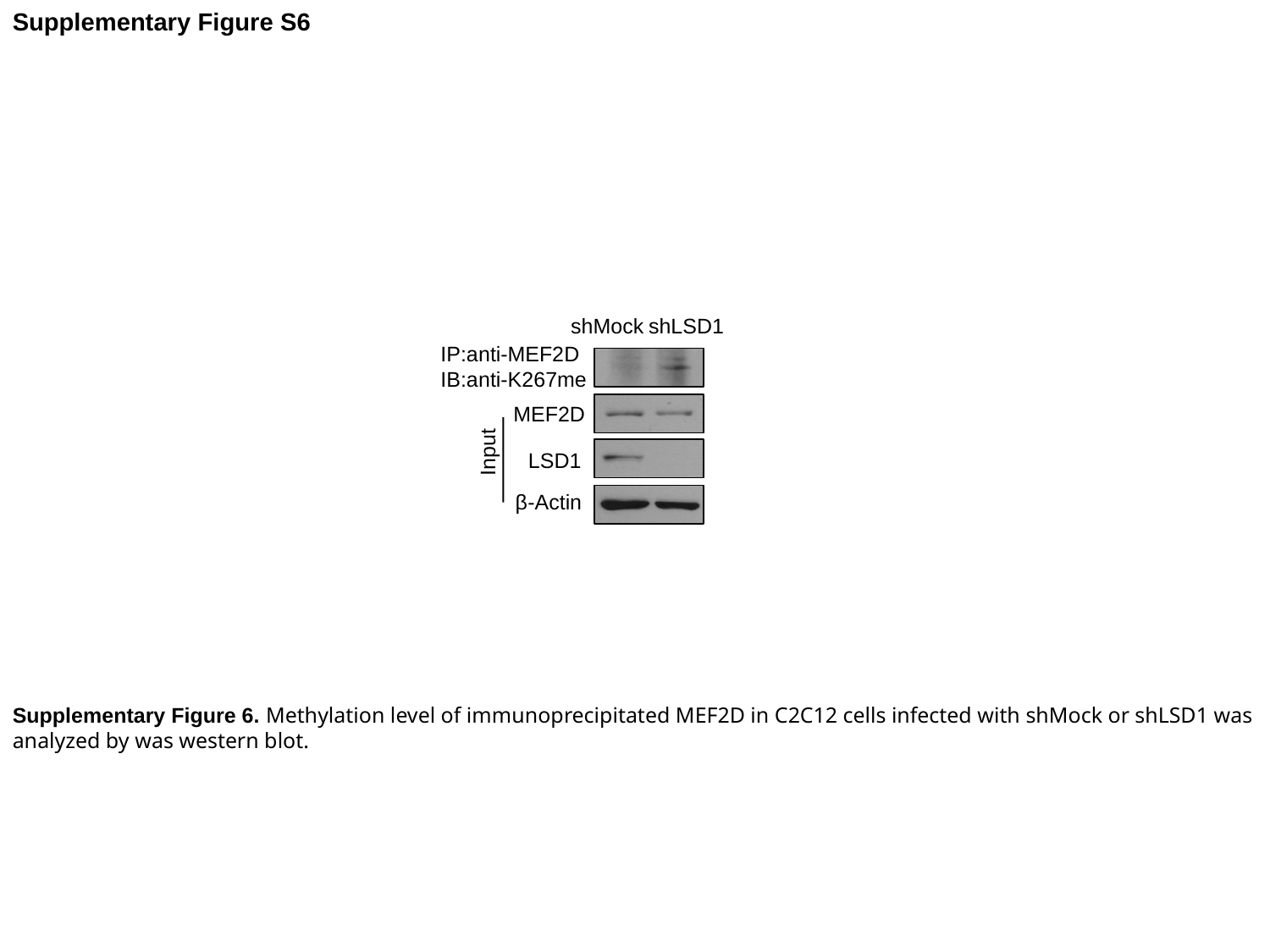

Supplementary Figure S6
shMock
shLSD1
IP:anti-MEF2D IB:anti-K267me
MEF2D
LSD1
Input
β-Actin
Supplementary Figure 6. Methylation level of immunoprecipitated MEF2D in C2C12 cells infected with shMock or shLSD1 was analyzed by was western blot.

## Slide 9
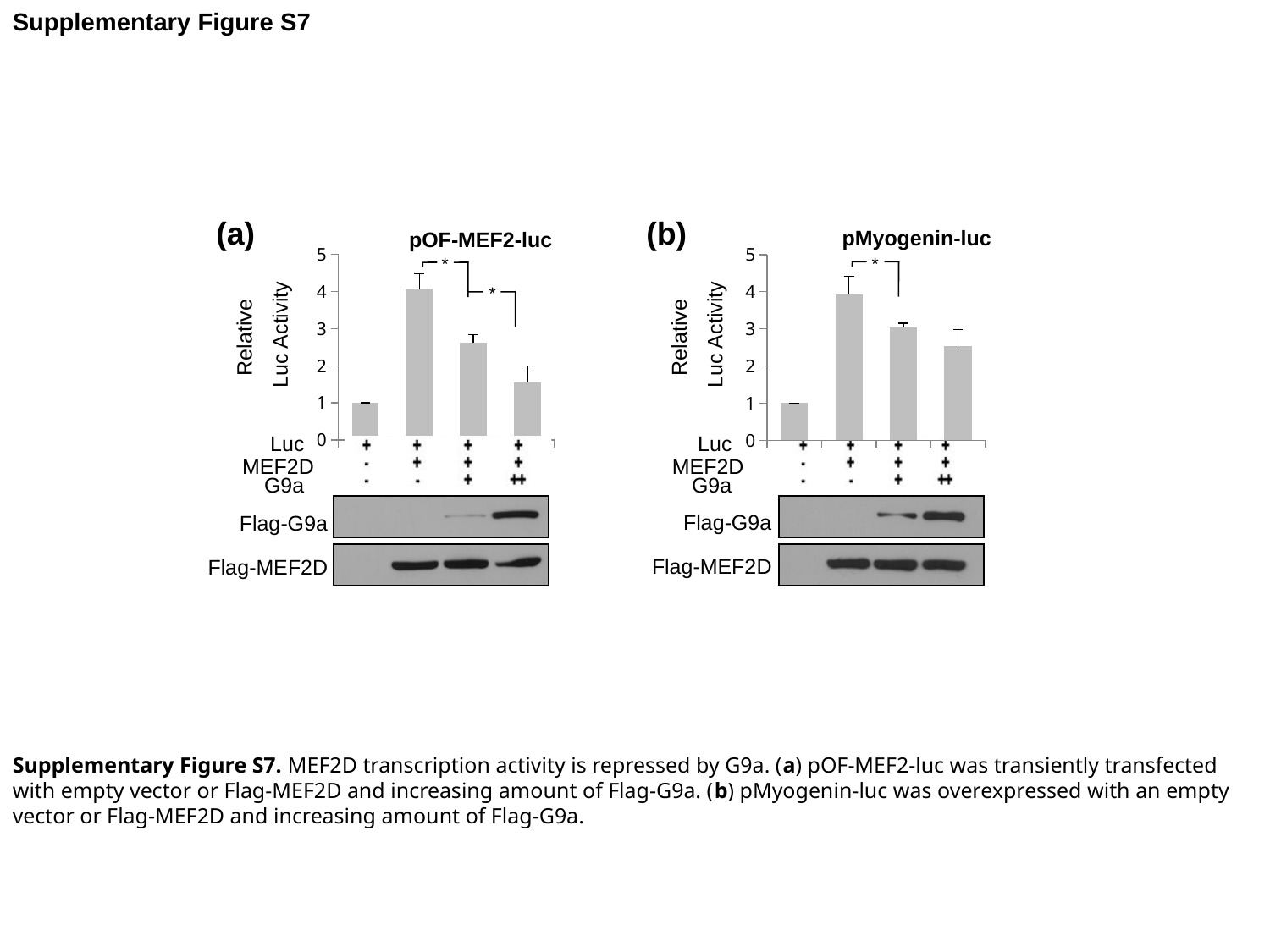

Supplementary Figure S7
(a)
(b)
pMyogenin-luc
pOF-MEF2-luc
Relative
Luc Activity
Relative
Luc Activity
### Chart
| Category | |
|---|---|
### Chart
| Category | |
|---|---|*
*
*
Luc
Luc
MEF2D
MEF2D
G9a
G9a
Flag-G9a
Flag-G9a
Flag-MEF2D
Flag-MEF2D
Supplementary Figure S7. MEF2D transcription activity is repressed by G9a. (a) pOF-MEF2-luc was transiently transfected with empty vector or Flag-MEF2D and increasing amount of Flag-G9a. (b) pMyogenin-luc was overexpressed with an empty vector or Flag-MEF2D and increasing amount of Flag-G9a.

## Slide 10
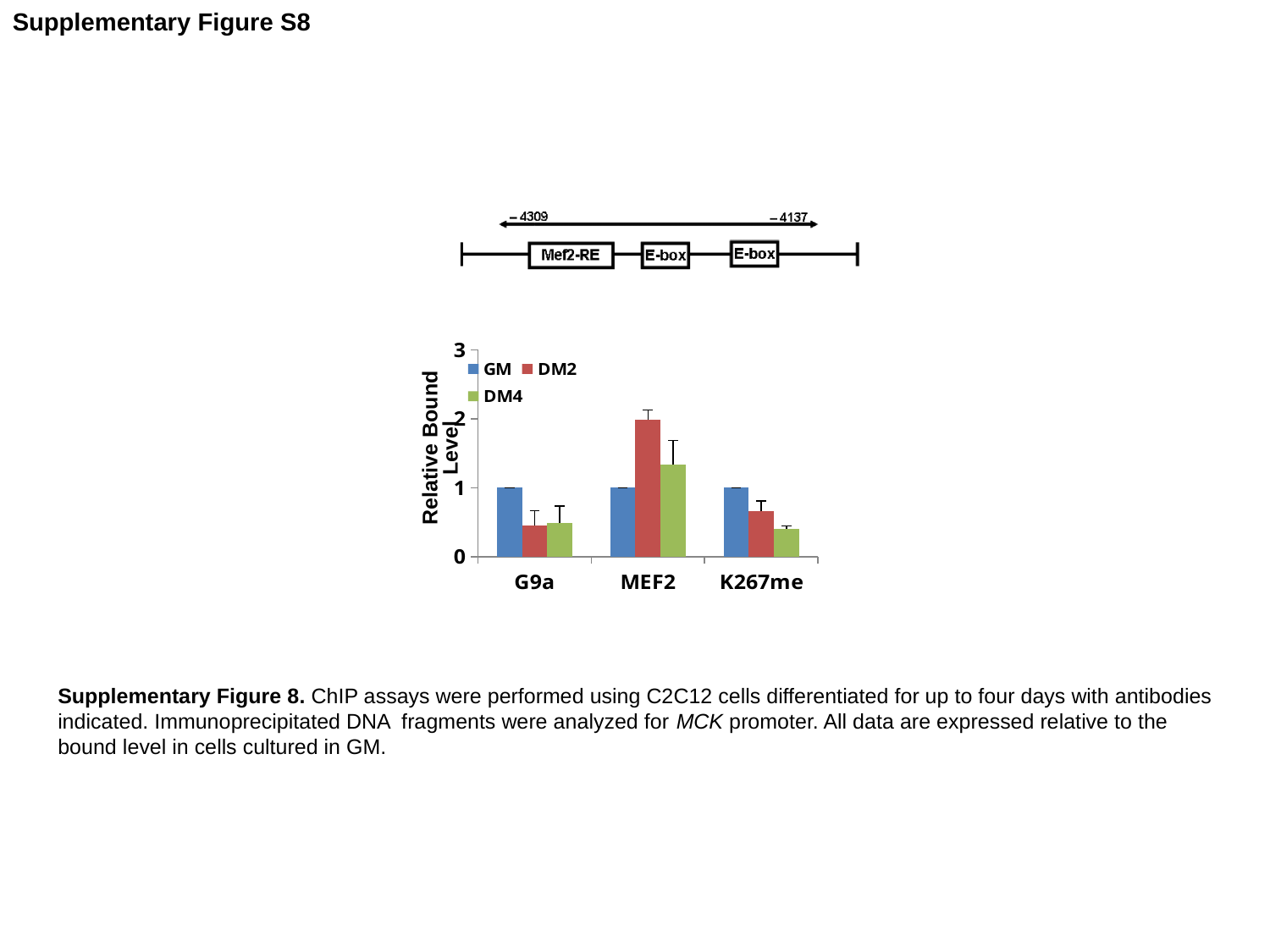

Supplementary Figure S8
Relative Bound Level
### Chart
| Category | GM | DM2 | DM4 |
|---|---|---|---|
| G9a | 1.0 | 0.44704365523124767 | 0.4837700293613375 |
| MEF2 | 1.0 | 1.980598603876404 | 1.3307917775686078 |
| K267me | 1.0 | 0.6558424658084852 | 0.40202792800459597 |Supplementary Figure 8. ChIP assays were performed using C2C12 cells differentiated for up to four days with antibodies indicated. Immunoprecipitated DNA fragments were analyzed for MCK promoter. All data are expressed relative to the bound level in cells cultured in GM.

## Slide 11
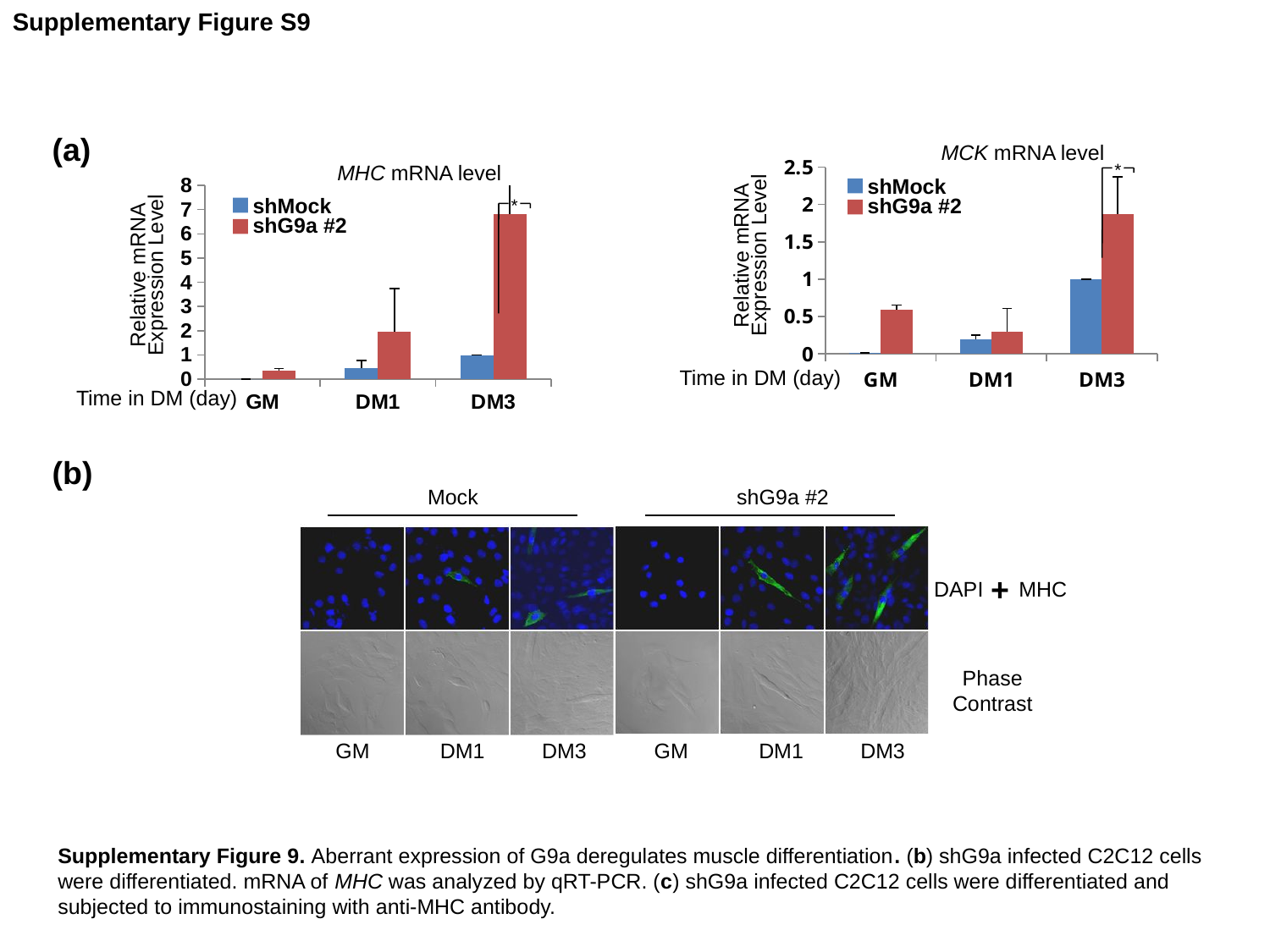

Supplementary Figure S9
(a)
MCK mRNA level
*
MHC mRNA level
### Chart
| Category | | |
|---|---|---|
| GM | 0.012828467314341148 | 0.5915586777855452 |
| DM1 | 0.1939681462065727 | 0.2988522853584664 |
| DM3 | 1.0 | 1.8722332739657161 |shMock
### Chart
| Category | | |
|---|---|---|
| GM | 0.0 | 0.3433504658896465 |
| DM1 | 0.460043545123705 | 1.94490781546419 |
| DM3 | 1.0 | 6.830978818793109 |
shMock
shG9a #2
*
shG9a #2
Relative mRNA
Expression Level
Relative mRNA
Expression Level
Time in DM (day)
Time in DM (day)
(b)
Mock
shG9a #2
+
DAPI MHC
Phase Contrast
GM
DM1
DM3
GM
DM1
DM3
Supplementary Figure 9. Aberrant expression of G9a deregulates muscle differentiation. (b) shG9a infected C2C12 cells were differentiated. mRNA of MHC was analyzed by qRT-PCR. (c) shG9a infected C2C12 cells were differentiated and subjected to immunostaining with anti-MHC antibody.

## Slide 12
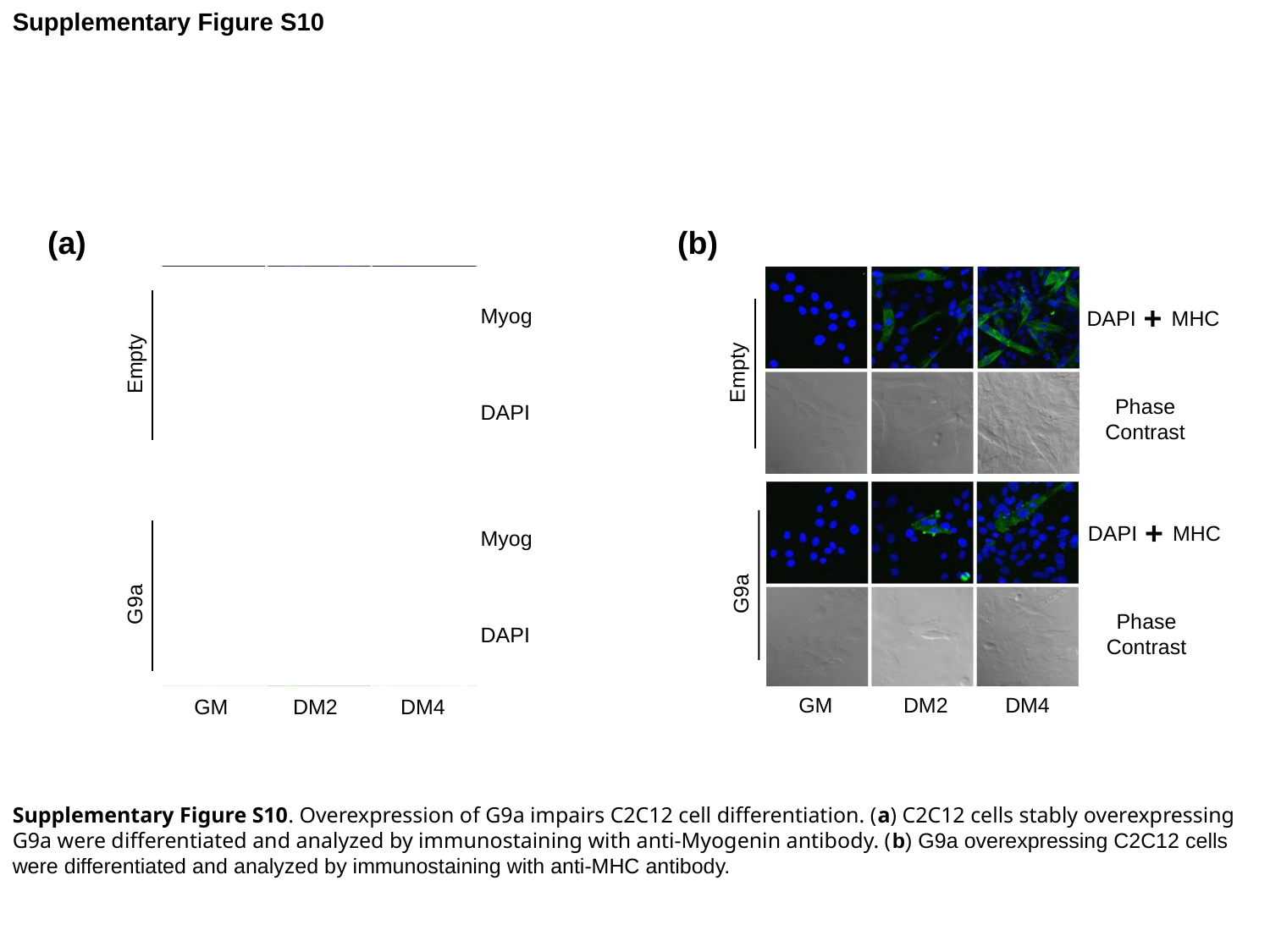

Supplementary Figure S10
(a)
(b)
+
Myog
DAPI MHC
Empty
Empty
Phase Contrast
DAPI
+
DAPI MHC
Myog
G9a
G9a
Phase Contrast
DAPI
GM
DM2
DM4
GM
DM2
DM4
Supplementary Figure S10. Overexpression of G9a impairs C2C12 cell differentiation. (a) C2C12 cells stably overexpressing G9a were differentiated and analyzed by immunostaining with anti-Myogenin antibody. (b) G9a overexpressing C2C12 cells were differentiated and analyzed by immunostaining with anti-MHC antibody.

## Slide 13
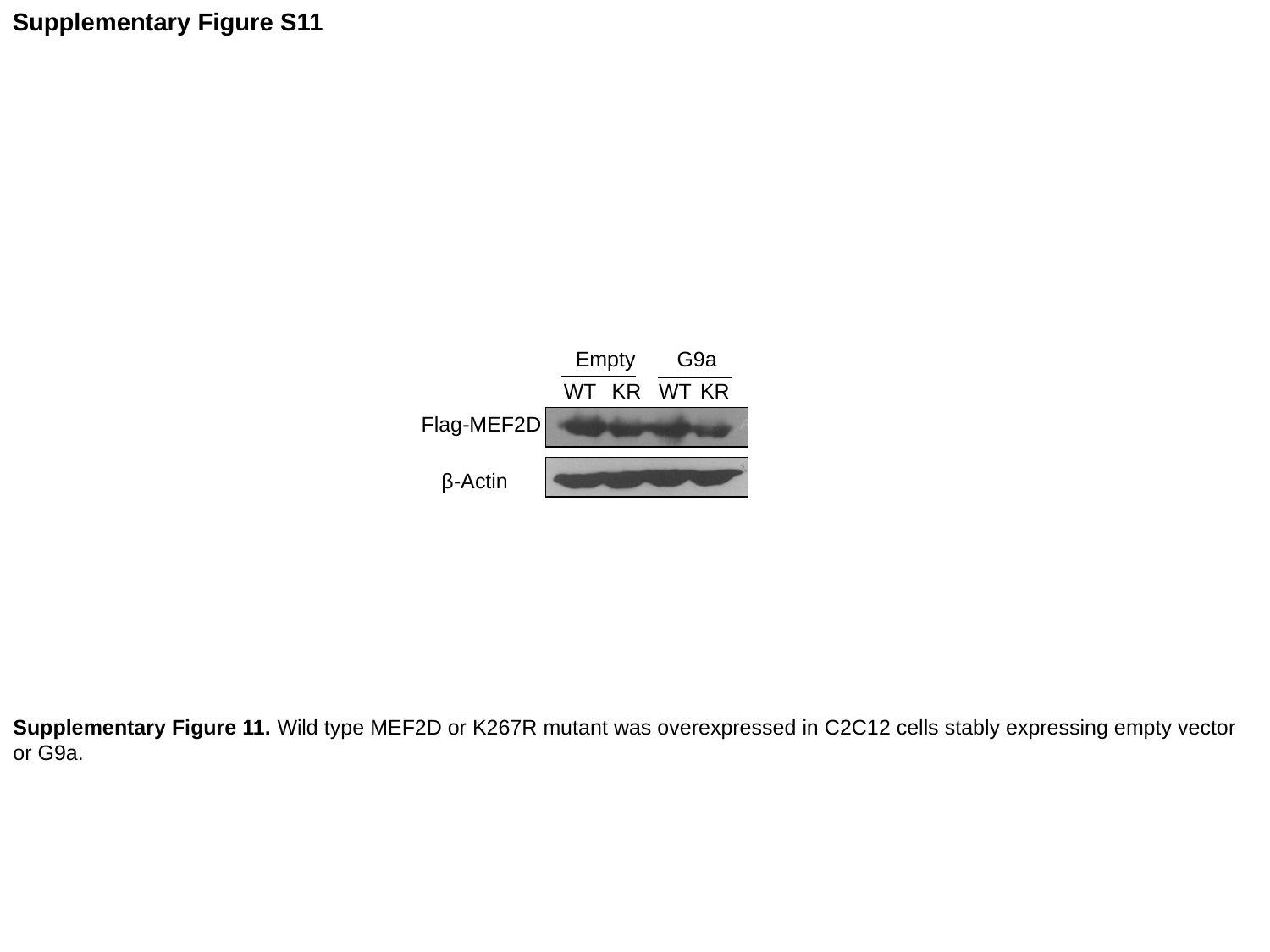

Supplementary Figure S11
Empty
G9a
KR
WT
KR
WT
Flag-MEF2D
β-Actin
Supplementary Figure 11. Wild type MEF2D or K267R mutant was overexpressed in C2C12 cells stably expressing empty vector or G9a.

## Slide 14
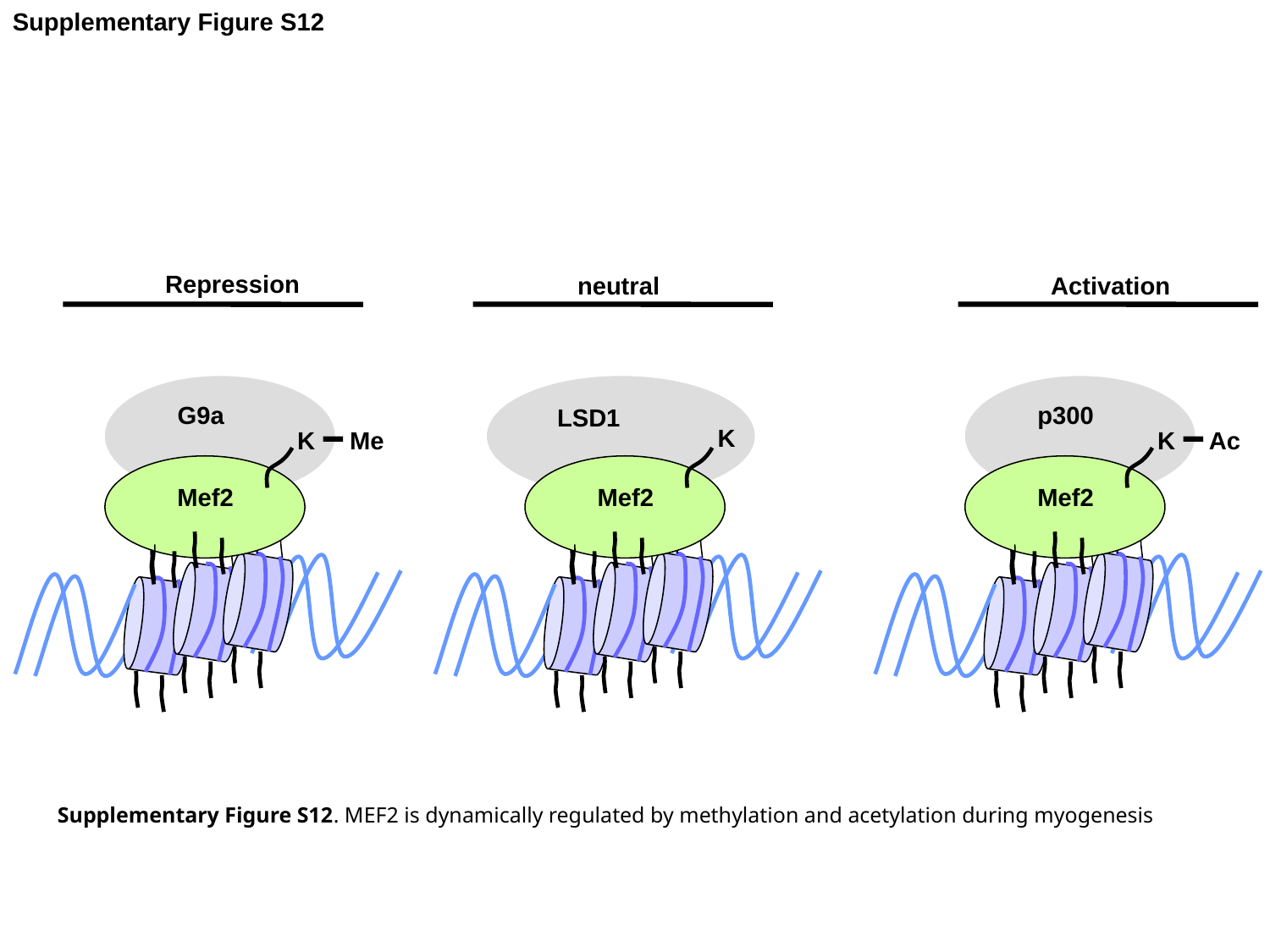

Supplementary Figure S12
Repression
neutral
Activation
LSD1
Mef2
G9a
p300
K
K Me
K Ac
Mef2
Mef2
Supplementary Figure S12. MEF2 is dynamically regulated by methylation and acetylation during myogenesis

## Slide 15
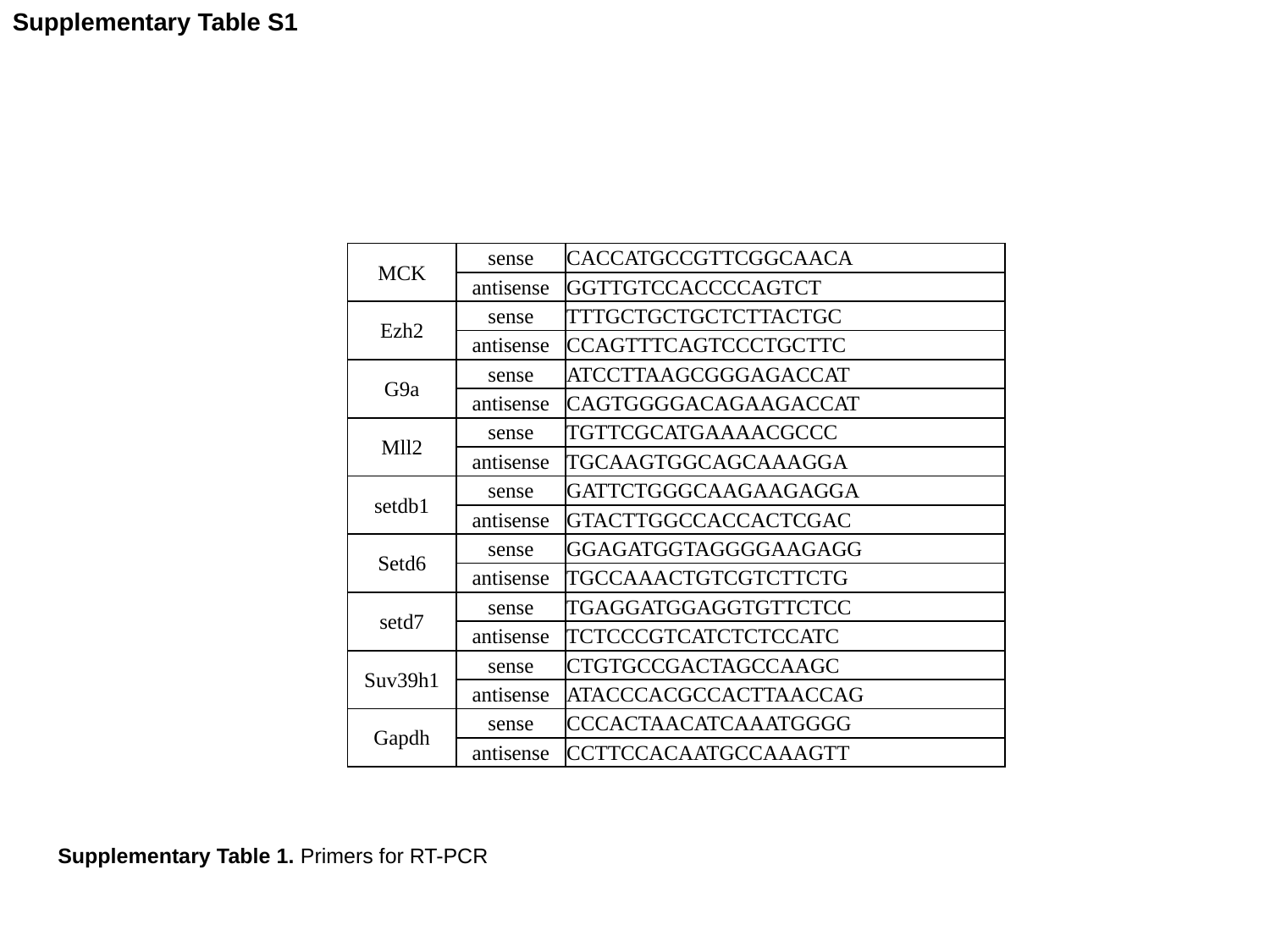

Supplementary Table S1
| MCK | sense | CACCATGCCGTTCGGCAACA |
| --- | --- | --- |
| | antisense | GGTTGTCCACCCCAGTCT |
| Ezh2 | sense | TTTGCTGCTGCTCTTACTGC |
| | antisense | CCAGTTTCAGTCCCTGCTTC |
| G9a | sense | ATCCTTAAGCGGGAGACCAT |
| | antisense | CAGTGGGGACAGAAGACCAT |
| Mll2 | sense | TGTTCGCATGAAAACGCCC |
| | antisense | TGCAAGTGGCAGCAAAGGA |
| setdb1 | sense | GATTCTGGGCAAGAAGAGGA |
| | antisense | GTACTTGGCCACCACTCGAC |
| Setd6 | sense | GGAGATGGTAGGGGAAGAGG |
| | antisense | TGCCAAACTGTCGTCTTCTG |
| setd7 | sense | TGAGGATGGAGGTGTTCTCC |
| | antisense | TCTCCCGTCATCTCTCCATC |
| Suv39h1 | sense | CTGTGCCGACTAGCCAAGC |
| | antisense | ATACCCACGCCACTTAACCAG |
| Gapdh | sense | cccactaacatcaaatgggg |
| | antisense | ccttccacaatgccaaagtt |
Supplementary Table 1. Primers for RT-PCR
